# Whole-Exome Sequencing of 21 Families: Candidate Genes for Early-Onset High Myopia

**DOI:** 10.3390/ijms242115676

**Published:** 2023-10-27

**Authors:** Eloísa Sánchez-Cazorla, Carmen González-Atienza, Ana López-Vázquez, Natalia Arruti, María Nieves-Moreno, Susana Noval, Rocío Mena, Carmen Rodríguez-Jiménez, Patricia Rodríguez-Solana, Eva González-Iglesias, Marta Guerrero-Carretero, Oriana D’Anna Mardero, Javier Coca-Robinot, Juan Carlos Acal, Joana Blasco, Carlos Castañeda, Jesús Fraile Maya, Ángela Del Pozo, María V. Gómez-Pozo, Victoria E. F. Montaño, Lucía De Dios-Blázquez, Carlos Rodríguez-Antolín, María de Los Ángeles Gómez-Cano, Luna Delgado-Mora, Elena Vallespín

**Affiliations:** 1 Molecular Ophthalmology Section, Medical and Molecular Genetics Institute (INGEMM) IdiPaz, Hospital Universitario La Paz, 28046 Madrid, Spain; eloisasancaz@gmail.com (E.S.-C.); carmenglezatienza@gmail.com (C.G.-A.); mariarocio.mena@salud.madrid.org (R.M.); crodriguezj@salud.madrid.org (C.R.-J.); prsolana@salud.madrid.org (P.R.-S.); eva.gonz.igl@gmail.com (E.G.-I.); mgdelpozo@salud.madrid.org (M.V.G.-P.); victoriaeugeniafdezmontano@hotmail.com (V.E.F.M.); 2 Department of Ophthalmology, IdiPaz, Hospital Universitario La Paz, 28046 Madrid, Spain; analopezvazquez94@gmail.com (A.L.-V.); natalia.arruti@salud.madrid.org (N.A.); maria.nieves@salud.madrid.org (M.N.-M.); susana.noval@salud.madrid.org (S.N.); martu_gue@hotmail.com (M.G.-C.); ori_odm86@hotmail.com (O.D.M.); cocajavi@hotmail.com (J.C.-R.); juancarlos.acal@salud.madrid.org (J.C.A.); yoana.blasco@yahoo.es (J.B.); carlos.castaneda@salud.madrid.org (C.C.); jesus.fraile@salud.madrid.org (J.F.M.); 3 European Reference Network on Eye Diseases (ERN-EYE), Hospital Universitario La Paz, 28046 Madrid, Spain; 4 Biomedical Research Center in the Rare Diseases Network (CIBERER), Carlos II Health Institute (ISCIII), 28029 Madrid, Spain; ingemm.adelpozo@gmail.com; 5 Clinical Bioinformatics Section, Medical and Molecular Genetics Institute (INGEMM) IdiPaz, CIBERER, Hospital Universitario La Paz, 28046 Madrid, Spain; ingemm.ldedios@gmail.com (L.D.D.-B.); crodrigueza@salud.madrid.org (C.R.-A.); 6 Clinical Genetics Section, Medical and Molecular Genetics Institute (INGEMM) IdiPaz, CIBERER, Hospital Universitario La Paz, 28046 Madrid, Spain; mariadelosangeles.gomez@salud.madrid.org (M.d.L.Á.G.-C.); lunadelde@gmail.com (L.D.-M.)

**Keywords:** early-onset high myopia, whole-exome sequencing, ophthalmogenetics, polygenic inheritance, candidate genes

## Abstract

High myopia is the most severe and pathological form of myopia. It occurs when the spherical refractive error exceeds –6.00 spherical diopters (SDs) or the axial length (AL) of the eye is greater than 26 mm. This article focuses on early-onset high myopia, an increasingly common condition that affects children under 10 years of age and can lead to other serious ocular pathologies. Through the genetic analysis of 21 families with early-onset high myopia, this study seeks to contribute to a better understanding of the role of genetics in this disease and to propose candidate genes. Whole-exome sequencing studies with a panel of genes known to be involved in the pathology were performed in families with inconclusive results: 3% of the variants found were classified as pathogenic, 6% were likely pathogenic and the remaining 91% were variants of uncertain significance. Most of the families in this study were found to have alterations in several of the proposed genes. This suggests a polygenic inheritance of the pathology due to the cumulative effect of the alterations. Further studies are needed to validate and confirm the role of these alterations in the development of early-onset high myopia and its polygenic inheritance.

## 1. Introduction

**Figure 1:**
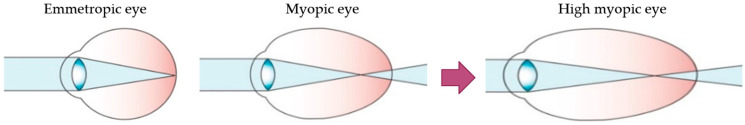
Schematic optics of the eye. In emmetropic eyes, the parallel rays of a distant object are focused precisely on the photoreceptors located on the retina. Myopic eyes have longer axial length, and the image of a distant object falls in front of the photoreceptors and cannot be brought into focus by accommodation. The myopic eye can become highly myopic, accentuating this defocus considerably. Modified from Morgan et al. [5].

Myopia is the most common eye disorder in the world [1]. From a physiological point of view, it involves a refractive error where the light rays entering the eye focus on a point in front of the retina, leading to decreased visual acuity. This refractive error is due to a postnatal axial elongation of the eye in the process of emmetropization [2,3]. Although myopia is usually considered a benign condition that can be corrected with the use of glasses, contact lenses or refractive surgery, it is becoming a public health concern due to its increasing prevalence in younger populations and its progression to its most severe and pathological form, high myopia (HM) (Figure 1). HM is defined as a spherical refractive error exceeding –6.00 spherical diopters (SD) or an axial length (AL) greater than 26 mm [4,5].

This study focuses on early-onset HM (EoHM), which occurs in children under 10 years of age [3]. Through an examination of the disease from a genetic perspective, this analysis seeks to contribute to a better understanding of the role of genetics in its development.

HM increases the susceptibility to other ocular complications that have a significant impact on quality of life and can lead to irreversible vision loss such as cataracts, glaucoma, retinal detachment and macular degeneration. It is therefore important to diagnose myopia and its progression to HM for early prevention and intervention before the onset of more severe pathological manifestations. In addition, HM can appear as one of the first clinical manifestations of conditions such as Marfan or Stickler syndromes, retinal disorders or congenital stationary night blindness (CSNB), allowing for the early diagnosis and treatment of these diseases [4].

**Figure 2:**
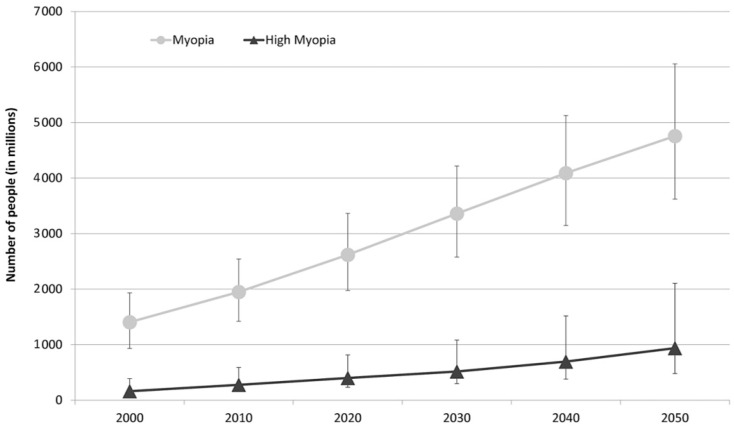
Graph representing the number of people estimated to develop myopia and HM for each decade from 2000 to 2050 [6].

The prevalence of myopia is highest in East Asia, affecting 90% of high school graduates. The reported prevalence of myopia among Caucasians in Europe is 30.6% and that of HM is 2.7%. Based on current trends, the study by Holden et al., estimates that the number of people affected will significantly increase worldwide by 2050, as may be observed in Figure 2 [4,6].

The etiology of myopia and HM is heterogeneous and multifactorial, involving a combination of environmental (external), microenvironmental (internal) and genetic factors [4].

Currently, the most studied environmental factor believed to protect individuals from developing myopia is the time spent outdoors [4]. Conversely, several studies have linked the progression of myopia to the time spent in near work and exposure to artificial light [3,5]. In the case of EoHM, however, given the young age of the patients, environmental factors are not considered to have a significant influence on the development of myopia. This suggests that there is a greater genetic burden involved in EoHM, supporting a genetic approach to the disease [3].

Microenvironmental factors, such as oxidative stress, oxidation and angiogenesis, can induce or accentuate EoHM. Given its high oxygen demand and direct exposure to natural light, the retina is particularly vulnerable to oxidative stress. In addition, patients with EoHM have an imbalance in the oxidative/antioxidative status of the retina, suggesting that oxidative stress plays a direct role in the development of this pathology [3].

**Figure 3:**
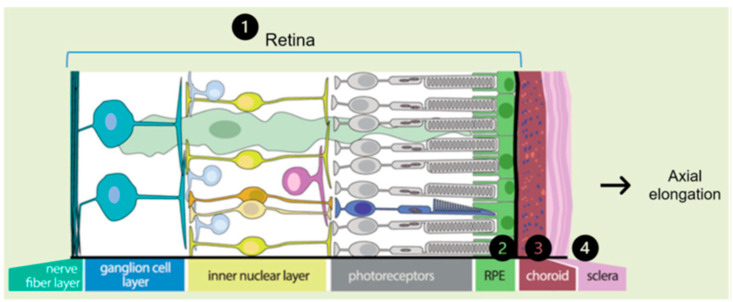
Representation of the multilayer signaling cascade that enables axial elongation. The signaling cascade order is represented by the numbers 1 to 4 Modified from Tedja et al. [7].

Genetic factors, as explained above, play a critical and major role in the development of EoHM. Genetic information initiates and regulates the processes involved in emmetropization, including the regulation of microenvironmental factors and visual feedback:

- Regulation of microenvironmental factors such as the level of angiogenic growth factors (VEGF, MCP1 and IL5) or proinflammatory cytokines (IL6, IFN-γ, IP-10, eotaxin and MIP-1α) in the aqueous and vitreous humors [3].
- Visual feedback driven by optical defocus. The defocus signal is detected by the retina and triggers a multilayered signaling cascade involving a large number of coding and regulatory genes, sequentially affecting the retina, retinal pigment epithelium (RPE), choroid, sclera and its extracellular matrix (ECM). In this last step of the cascade, changes occur in the composition of the ECM and are reflected in axial elongation (Figure 3). Alterations in these genes and their function can disrupt emmetropization and result in excessive axial length [2].

The classification of genetic alterations affecting visual feedback may be divided into two main groups: those involved in the retina, affecting the function of photoreceptors, RPEs, and ON and OFF bipolar neurons; and those that occur in the sclera, affecting the composition and metabolism of the ECM [5].

The role of genetic factors in myopia is supported by several studies. It has been observed that children with a family history of myopia have longer axial lengths and a higher risk of developing myopia during childhood compared to the average population [4]. Due to differences in study design and methods of analysis, heritability estimates in the literature range from 15% to 98%; however, the true heritability of myopia is probably between 60% and 80%.

Genome-wide association studies (GWASs) and segregation analyses have identified more than 26 loci and 400 genes involved in refractive error and the development of common myopia [4,7]. Fewer genes have been associated with EoHM: 11 genes with autosomal dominant inheritance have been identified for its non-syndromic form (*ZNF644*, *SCO2*, *SLC39A5*, *CCDC111*, *P4HA2*, *BSG*, *CPSF1*, *NDUFAF7*, *TNFRSF21*, *XYLT* and *DZIP1*); four genes with autosomal recessive inheritance (*LRPAP1*, *CTSH*, *LEPREL1* and *LOXL3*); and two X-linked genes (*ARR3* and *OPN1LW*). Other studies have associated EoHM with loci involved in the development of common myopia and refractive error, as well as with other genes such as *CTNND2*, *JOANA*, *CACNA1F*, *RPGR*, *PRSS56*, *BMP3*, *KCNQ5*, *LAMA2*, *TOX*, *TJP2*, *RDH5*, *ZIC2*, *RASGRF1*, *GJD2*, *RBFOX1*, *SHISA6*, *FAM150B-ACP1*, *LINC00340*, *FBN1*, *DIS3L-MAP2K1*, *ARID2-SNAT1* and *SLC14A2* [3].

Most of the variants studied, especially in common myopia, carry a low risk and can be prevalent in the general population, contributing to a small extent and cumulatively to the overall risk. Therefore, it is postulated that the inheritance of myopia and high myopia follows a polygenic pattern, in which several genes together contribute to the manifestation of the disease [8].

In this study, next-generation sequencing (NGS) was used to sequence the whole exome of 21 families diagnosed with EoHM. Whole-exome sequencing makes it possible to study alterations and their involvement in a larger number of genes, thus allowing for the diagnosis and proposal of new candidate causal genes for EoHM in patients.

## 2. Results and Discussion

The results of this study were obtained from an ongoing project seeking to identify new genes responsible for EoHM in a sample of families from a tertiary hospital in Spain. This project also aims to evaluate the implementation of NGS and its relevance to the management of patients with EoHM. A total of 21 probands (33.33% female [7/21] and 66.66% male [14/21]) and nine affected relatives with EoHM from 21 unrelated families were recruited based on their phenotype (Table 1) and the inclusion criteria indicated in the Materials and Methods.

**Figure 4:**
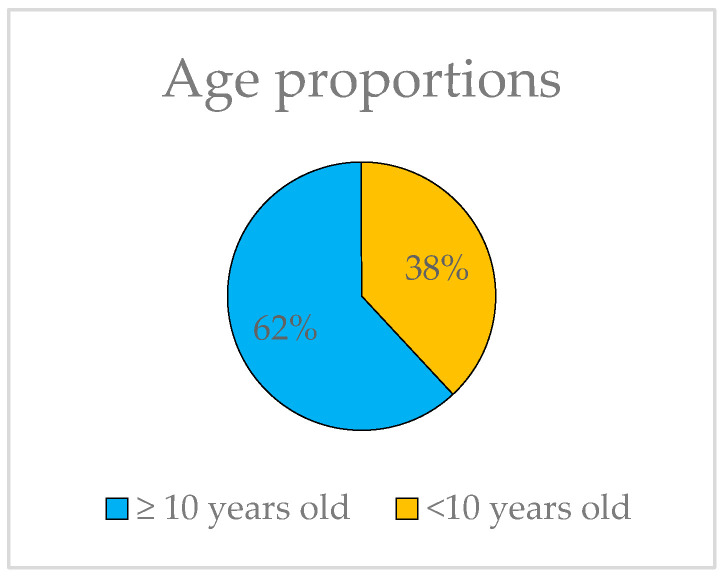
Representation of the proportions of probands younger and older than 10 years old.

The ages of the probands range from 6 to 80, yet they all share the common characteristic of exhibiting HM before the age of 10, thus constituting a unified group for genetic analysis. It is worth noting that 62% of the subjects in this study have not yet reached the age of 10 (Figure 4).

The average spherical equivalent for the probands is −12.044 diopters in the right eye and −11.499 diopters in the left eye. Table 2 shows the descriptive statistics, including the mean and standard deviation of the refractive outcomes in this cohort.

**Figure 5:**
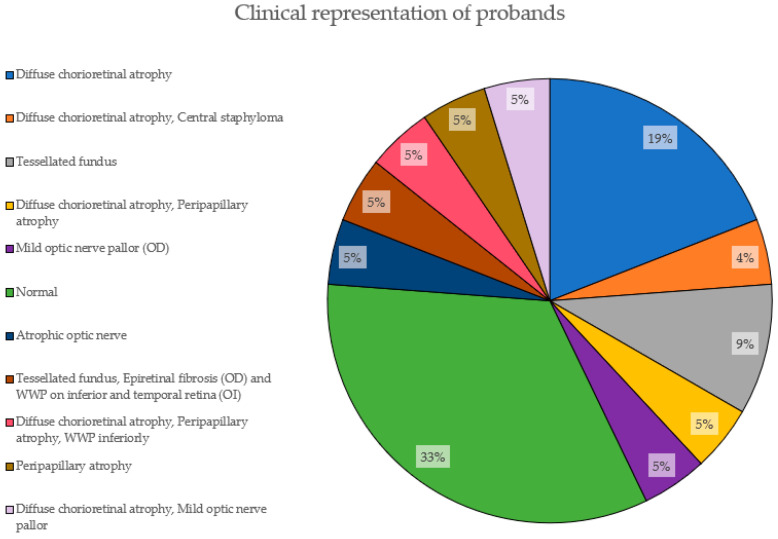
Clinical representation of probands. The proportions of features presented by the probands in this study are shown here, with their corresponding percentage.

Figure 5 depicts the clinical characteristics of the EoHM patients based on fundoscopic examination. 33% of them had not developed yet any features (normal). The most prevalent phenotypic feature is diffuse chorioretinal atrophy, present in 19% of the cases alone and adding a total of 38% accompanied by other features, followed by a tesselated fundus, with 9%.

For the gender-based difference analysis, the patients were divided into two groups: more severe EoHM and less severe EoHM, obtaining the following frequencies (Table 3).

Since we have frequencies lower than 5, a Fisher’s exact test was performed, resulting in a *p*-value of 0.6557. Given that this *p*-value is greater than 0.05, there is insufficient evidence to reject the null hypothesis (it can be found in Materials and Methods section). Thus, there is no association between severity and gender; they are independent variables.

The results obtained were evaluated considering the limitations of exome sequencing such as the low capture efficiency in the sequencing of certain regions, the inability to analyze regulatory regions far from the exons, the lack of previous scientific studies of yet unknown genes and the inability to conduct functional studies of all the variants found.

As one of the objectives of this work was to identify new genes responsible for EoHM and exome sequencing implies the possibility of finding variants in genes not previously related to ocular pathology, we considered variants classified as pathogenic (P), likely pathogenic (LP) and variants of uncertain significance (VUS) not previously associated with HM, but that are involved in the regulation of emmetropization.

**Figure 6:**
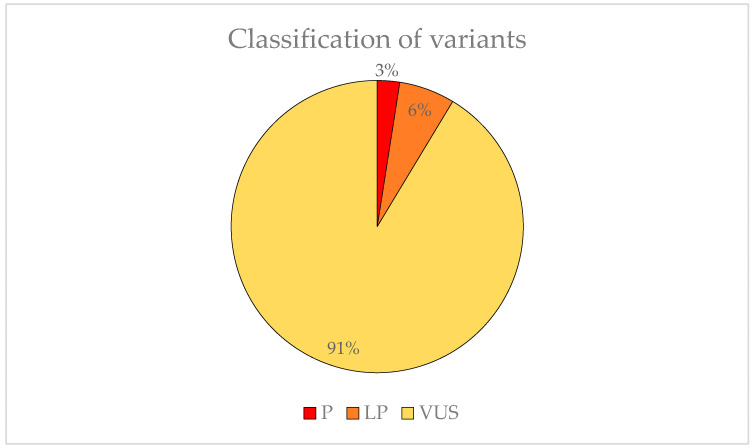
Representation of the variant pathogenicity found in possible candidate genes for EoHM.

We found 83 variants that may be involved in the development of EoHM. Two (3%) were classified as P, five (6%) was LP and 74 (91%) was VUS (Figure 6). The American College of Medical Genetics (ACMG) criteria for classifying pathogenic variants may be observed in Table A1.

The alterations found in the families included in the study and their relationship to EoHM are detailed below.

### 2.1. OFT-00074 Family

Candidate genes on Table 4. One of the proposed causal alterations for EoHM in this family was classified as pathogenic and affects the splicing of the *TRPM1* gene. Its associated pathology is night blindness, congenital stationary (complete), 1C and autosomal recessive [9]. It encodes a permeable calcium cation channel, mainly expressed in retinal bipolar cells. Because CSNB and EoHM are associated and cases of EoHM have been observed in CSNB patients with altered *TRPM1*, the involvement of this gene in its development has been proposed, although the cause of this association is not yet known [10,11,12]. It has been hypothesized that *TRPM1* plays a role in embryonic development by influencing synaptic activity, optic nerve formation, photoreceptor arrangement and ON bipolar cell function [13]. In the Gene Ontology Resource database, this gene is associated with visual perception and the cellular response to light stimulus (Table A2) [14].

Other VUS alterations detected in this family included that of *ARHGEF18*, which codes for a guanine nucleotide exchange factor (GEF) that directly controls the activation of Rho GTPases and is involved in retinal development and degeneration [15]. This gene is a key regulator of RhoA-Rock2 signaling, which is crucial for the maintenance of polarity in the vertebrate retinal epithelium. In addition, *ARHGEF18* is required to maintain apico-basal polarity, tight junction localization and cortical actin, thus shaping the morphology of these cells [16]. This gene is associated with retinitis pigmentosa 78 [9].

*KDM6B* encodes a lysine-specific demethylase that demethylates histone H3 (epigenetic control) and other non-histone proteins. It is involved in processes of organogenesis and retinal development at the amacrine, horizontal and ganglion cell level, allowing the focusing or defocusing signal to correctly reach the brain from the retina [17].

The selected candidate genes did not have AD inheritance and were not found in homozygosity, suggesting the possibility of a cumulative effect of the proposed alterations as a cause of EoHM in the affected individuals.

**Table 4:** Candidate genes of the OFT-00074 family.

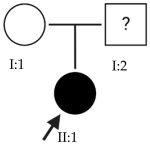	ACMG Criteria	ACMG Result	Variant	Zygosity	Inheritance	Total Families	Model of Inheritance	Gene Reported by
*ARHGEF18*	PM2	VUS	NM_015318.3:c.2167C>T:p.(Arg723Cys)	Het	Unknown	1	AR	[16]
*KDM6B*	PM2, BP4	VUS	NM_001080424.1:c.1582C>T:p.(Pro528Ser)	Het	Unknown	2	Unknown	[17]
*TRPM1*	PVS1, PM2, PP5	P	NM_002420.5:c.1023+1G>A	Het	Unknown	2	AR	[10,11,12,13]

Het: heterozygous; AR: autosomal recessive.

### 2.2. OFT-00097 Family

Candidate genes on Table 5. The proband in this family had a variant inherited from his mother of the most altered gene identified in this study, *HSPG2*. This is a gene with incomplete penetrance [18], encoding perlecan, a large multidomain proteoglycan. It binds and cross-links to ECM components and molecules on the cell surface, allowing it to interact with laminin, prolargin, type IV collagen, FGFBP1, FBLN2, FGF7 and transthyretin and maintain the endothelial barrier function in the vascular ECM. *HSPG2* also promotes the activity of growth factors such as FGF2, thus stimulating endothelial growth and fibroblast regeneration [15]. The study by Wan et al., includes it as one of the new candidate genes for EoHM [19].

Other altered genes affecting the structure of the sclera were also observed, such as *COL9A2*, a collagen associated with Stickler syndrome, which, being a heterozygous variant, may have contributed to HM in conjunction with the other alterations without developing the full syndrome [20]. Fibulin1 (*FBLN1*) is a glycoprotein incorporated into a fibrillar ECM [15], which is expressed in fibroblasts of the sclera, enabling cell–matrix interactions, and is involved in the regulation of ocular growth [21].

*CACNA1F* presents a pathogenic alteration in homozygosis that adds a stop codon. The protein expressed is a multi-pass transmembrane that functions as an alpha-1 subunit of the voltage-dependent calcium channel and mediates the entry of calcium ions into the cell. *CACNA1F* is associated with several X-linked eye disorders including CSNB type 2A and Aland Island eye disease [15]. The gene is involved in the cone and rod response and was identified in a study of patients with CSNB as predominant in those who also had EoHM [22].

*CSMD1*, another of the most altered genes found in this study, is located at the MYP10 locus and is mainly expressed in the peripheral retina and in the area surrounding the macula. This gene plays a critical role in cone growth, including signal transduction and matrix adhesion, and has been proposed in multiple studies as a candidate gene for the development of EoHM [19,23].

Finally, an alteration in *ADAMTSL1* is proposed as possibly contributing to the development of EoHM by encoding a thrombospondin motif metalloproteinase (ADAMTS). This protein may play important roles in the ECM, thus establishing a link to myopia [15].

In the case of this family, the most relevant alterations that may have influenced the EoHM of the probands are those present in the *HSPG2*, *FBLN1* and *CACNA1F* genes, due to their inheritance pattern, although the other genes mentioned may also have contributed. Specifically, *CSMD1* has been observed and proposed in this study and other related studies [19,23].

**Table 5:** Candidate genes of the OFT-00097 family.

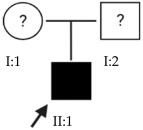	ACMG Criteria	ACMG Result	Variant	Zygosity	Inheritance	Total Families	Model of Inheritance	Gene Reported by
*ADAMTSL1*	PM2	VUS	NM_001040272.5:c.1819G>A:p.(Glu607Lys)	Het	Unknown	1	Unknown	[15]
*CACNA1F*	PVS1, PM2, PP5	P	NM_005183.3:c.4504C>T:p.(Arg1502 *)	Hemi	Maternal	2	XL	[22,24]
*COL9A2*	PM2	VUS	NM_001852.3:c.1652C>T:p.(Ala551Val)	Het	Unknown	1	AR	[20]
*CSMD1*	PM2	VUS	NM_033225.5:c.1712A>G:p.(Asn571Ser)	Het	Maternal	4	Unknown	[19,23]
*FBLN1*	PM2	VUS	NM_006486.2:c.1157C>T:p.(Thr386Met)	Het	Unknown	1	AD	[21]
*HSPG2*	PM2	VUS	NM_005529.6:c.12691G>A:p.(Glu4231Lys)	Het	Maternal	6	AD/AR	[19]

Het: heterozygous; Hemi: hemizygous; AD: autosomal dominant; AR: autosomal recessive; XL: X-linked. * Gain of a stop codon, does not substitute for any amino acid.

### 2.3. OFT-00155 Family

Candidate genes on Table 6. In this family, we searched for alterations common to the two affected individuals, as well as including other genes, also shared with the unaffected sibling, reported in studies of EoHM or myopia. All the altered genes are involved in the maintenance of the sclera, with the exception of *CACNA1F*, as explained above, and *COL9A3*, a collagen described in Stickler syndrome, in which the most involved ocular structure is the choroid [15]. The role of *HSPG2* in the development of EoHM has been detailed above.

The *LAMA1* gene is at the MYP2 locus and belongs to a family of structural glycoproteins in the ECM of the sclera and lens [15,25]. It is one of the genes most associated with the development of EoHM, supported by several studies [25,26,27]. Because it has an AR inheritance and is heterozygous, it is likely not the only altered gene causing EoHM in this family. There could be a cumulative effect enhanced by the other alterations indicated in Table 4, as most of these affect the sclera. For example, an alteration was observed in *LAMA5*, another glycoprotein in basement membranes with biological functions similar to *LAMA1* [15].

Alterations were also found in the *THBS2* and *THBS1* genes, homotrimeric-disulfide-linked glycoproteins that mediate cell–cell and cell–matrix interactions. *THBS1* can bind to fibrinogen, fibronectin, laminin, type-V collagen and alpha-V/beta-1 integrins [15]. In a study of patterns of messenger RNA (mRNA) in sclera remodeling during the development of lens-induced myopia in Soricidae, changes were observed in the *SPARC*, *THBS1*, *THBS2*, *TNC* and *SPP1* genes, suggesting that these may play a role in increasing scleral sliding velocity [28].

### 2.4. OFT-00175 Family

Candidate genes on Table 7. All the alterations found in the OFT-00175 family were classified as VUS. The proband had only one de novo alteration in *HSPG2*; the other alterations were inherited from his father.

The altered genes affecting the retina are *PCDH15* and *TRPM1*. *PCDH15* belongs to the cadherin superfamily, which encodes integral membrane proteins responsible for mediating calcium-dependent cell–cell adhesion. It plays an essential role in the maintenance of normal retinal and cochlear function [15]. *PCDH15* was proposed in the WES study by Wan et al., as a candidate gene related to EoHM [19], with an AR or digenic inheritance model. Digenic inheritance is that in which a mutation in two genes is necessary to cause a given phenotype. In this case, there were alterations in four genes.

Finally, *BMPR2* is a member of the bone morphogenetic protein (BMP) family of transmembrane serine/threonine kinase receptors. Ligands for this receptor are members of the transforming growth factor (TGF)-β superfamily [15]. This gene is also expressed in the retina but may have some relation to the development of EoHM given its association with the function of scleral fibroblasts in early myopia and incomplete penetrance [29].

**Table 7:** Candidate genes of the OFT-00175 family.

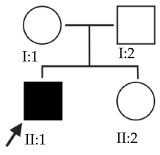	ACMG Criteria	ACMG Result	Variant	Zygosity	Inheritance	Total Families	Model of Inheritance	Gene Reported by
*BMPR2*	PM2	VUS	NM_001204.6:c.1931A>G:p.(Asn644Ser)	Het	Paternal	1	AD	[29]
*HSPG2*	PM2	VUS	NM_005529.6:c.10481G>A:p.(Arg3494Gln)	Het	*De novo*	6	AD/AR	[19]
*PCDH15*	PM2	VUS	NM_001142769.1:c.4396A>G:p.(Ser1466Gly)	Het	Paternal	2	AR/Digenic	[19]
*TRPM1*	BP4	VUS	NM_002420.5:c.4433C>T:p.(Thr1478Met)	Het	Paternal	2	AR	[10,11,12,13]

Het: heterozygous; AD: autosomal dominant; AR: autosomal recessive.

### 2.5. OFT-00178 Family

Candidate genes on Table 8. In this family, variants inherited from the mother and father were found affecting both scleral and retinal development, which could cumulatively cause EoHM.

Genes involved in the identified retinal development included *PCDH15* and *CSMD1*, discussed above. *LRP2*, another gene in this group, encodes a multi-ligand endocytic receptor, has a role in cell signaling and has been associated with Donnai–Barrow and Stickler syndromes, both of which are characterized by EoHM [15]. This gene was found to have a necessary function for normal eye growth through inactivation of Lrp2 in the mouse forebrain (including the neural retina and retinal and ciliary pigment epithelia), resulting in a 40% greater axial elongation compared to controls. Bipolar, photoreceptor and retinal ganglion cells were also affected [30].

The proband had two heterozygous alterations in the *MAP3K1* gene, one inherited from his mother and the other from his father, known as double heterozygous or compound heterozygous. This gene is known to be involved in eye development, with high expression in the retina. Its low expression in the mouse retina affects vascularization, RPE, photoreceptor loss and early degeneration [31,32].

*LAMA4* is another gene associated with alterations affecting the sclera. Expression changes in the HIF-1α/miR-150-5p/LAMA4/p38 MAPK axis have been observed in ECM degradation of scleral fibroblasts under hypoxic conditions, leading to the pathological progression of HM. An increased expression of *LAMA4* has been observed in patients with HM [33].

At the scleral level, excessive *PLG* expression has been reported in HM patients. Here, plasmin (in its active form) can degrade fibrin and convert potential matrix metalloproteinases (pro-MMPs) into active MMPs, capable of destroying the ECM, thus reducing scleral stiffness and making it unable to maintain its necessary stiffness, strength and elasticity. Plasmin also participates in other processes involved in the pathogenesis of HM such as tissue remodeling and angiogenesis [34].

*VASH1* enables actin-binding and metallocarboxypeptidase activity. It is involved in the negative regulation of angiogenesis, the migration of blood vessel endothelial cells and proteolysis [15] and is expressed in the neural retina, sclera, choroid and RPE [35].

Several of these alterations may play a significant role in EoHM.

**Table 8:** Candidate genes of the OFT-00178 family.

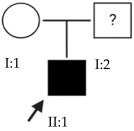	ACMG Criteria	ACMG Result	Variant	Zygosity	Inheritance	Total Families	Model of Inheritance	Gene Reported by
*CSMD1*	PM2	VUS	NM_033225.5:c.8042G>A:p.(Gly2681Asp)	Het	Paternal	4	Unknown	[19,23]
*CSMD1*	PM2, BP4	VUS	NM_033225.5:c.4375G>A:p.(Ala1459Thr)	Het	Paternal	4	Unknown	[19,23]
*LAMA4*	PM2	VUS	NM_001105207.2:c.673G>A:p.(Ala225Thr)	Het	Paternal	1	AD	[33]
*LRP2*	PM2, PP3	VUS	NM_004525.2:c.10202C>G:p.(Thr3401Arg)	Het	Maternal	1	AR	[30]
*MAP3K1*	PM2, BP4	VUS	NM_005921.1:c.299G>A:p.(Gly100Glu)	Het	Maternal	1	AD	[31,32]
*MAP3K1*	PM2, PM4	VUS	NM_005921.1:c.3646_3648delATC:p.(Ile1216del)	Het	Paternal	1	AD	[31,32]
*PCDH15*	PM2, BP4	VUS	NM_001142769.1:c.1519G>A:p.(Val507Ile)	Het	Paternal	2	AR/Digenic	[19]
*PLG*	PM2, PP3	VUS	NM_000301.3:c.598A>G:p.(Thr200Ala)	Het	Paternal	1	AR	[34]
*VASH1*	PM2	VUS	NM_014909.4:c.953G>A:p.(Arg318Gln)	Het	Maternal	1	Unknown	[35]

Het: heterozygous; AD: autosomal dominant; AR: autosomal recessive.

### 2.6. OFT-00191 Family

Candidate genes on Table 9. The *PER3* gene belongs to the period gene family, which encodes components of the circadian rhythms of locomotor activity, metabolism and behavior [15]. It has a role in the negative regulation of transcription by RNA polymerase II [14]. Circadian rhythm genes are associated with refractive error. *PER3* is located near the MYP14 locus and is expressed in ON and OFF bipolar cells [36].

*COL11A1*, also associated with Stickler and Marshall syndromes, may only present a phenotype in the eye, such as EoHM [15,37]. As for *FRMPD1*, the protein it encodes directly interacts with Gpsm2 (G-protein signaling modulator 2) and is necessary for the optimization of the rod-to-bipolar synaptic transmission when Gαt is present at the synapse [38].

In the two affected individuals of this family, the effect of the alteration in *COL11A1* predominates, making it the primary factor in the pathogenesis of the disease given its inheritance pattern, classification, extensive evidence in the literature and association with Stickler and Marshall syndromes.

**Table 9:** Candidate genes of the OFT-00191 family.

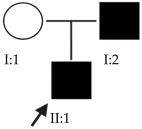	ACMG Criteria	ACMG Result	Variant	Zygosity	Inheritance	Total Families	Model of Inheritance	Gene Reported by
*COL11A1*	PP3, PM2	LP	NM_001854.3:c.2900G>T:p.(Gly967Val)	Het	Paternal	1	AD/AR	[15,24]
*FRMPD1*	PM2, BP4	VUS	NM_014907.2:c.2469C>A:p.(Ser823Arg)	Het	Paternal	2	Unknown	[38]
*PER3*	PM2, BP4	VUS	NM_016831.2:c.3502A>G:p.(Thr1168Ala)	Het	Paternal	1	AD	[36]

Het: heterozygous; AD: autosomal dominant; AR: autosomal recessive.

### 2.7. OFT-00209 Family

Candidate genes on Table 10. The candidate genes for EoHM in this family include *GLB1*. The protein it encodes (galactosidase beta 1) is present in human choroidal endothelial cells and acts as a receptor for elastin-derived peptides (EDPs). Elevated levels of circulating EDP do not affect retinal function in mice but increase the expression and deposition of collagen IV in the RPE/choroid complex [39]. *COL9A1* is associated with Stickler syndrome [15]; *KDM6B* has been discussed above.

### 2.8. OFT-00217 Family

Candidate genes on Table 11. The proband in family OFT-00217 inherited alterations from both her mother and father. *AGRN* is located at the MYP14 locus, encoding agrin, a large proteoglycan with multiple isoforms. It contains several laminin G domains, a Kazal-like serine protease inhibitor and epidermal growth factor. The study by Zheng et al. [40] found that *AGRN* is involved in baseline refractive development, demonstrating that this gene interacts with *EGR1*, which is implicated in refractive development and regulates synaptic physiology in the retina [15,40]. Several studies of different populations have associated rare or infrequent heterozygous alterations in *AGRN* with the development of HM [40].

The *GRM6* gene is involved in ON synaptic transmission in bipolar cells, controlling the release of dopamine, which has been suggested as a factor in ocular growth. Mutations in this gene cause CSNB type 1B. HM is often seen in CSNB type 1B patients with altered *GRM6*. A link between *GRM6* and susceptibility to HM has been suggested in a number of studies [41].

Alterations in the *CNTN6* gene may be involved in the development of EoHM, as contactin-6 is responsible for the correct development and maintenance of the central nervous system, particularly in axonal projection, branching and synapses. Meguro et al., associated an altered expression of this gene affecting GABA receptor levels or synaptogenesis with HM [42]. *FRMD4B* is also involved at the retinal level, through direct interaction of the protein it encodes with cytohesin-3, and functions as a scaffolding protein. Cytohesin-3 plays an important role in insulin, epidermal and nerve growth factor signaling, with a recent study [42] suggesting that the FRMD4B-cytohesin-3 complex affects cell junction dynamics in the retina and contributes to the regulation of photoreceptor cell growth and development [42].

*LRP1* affects the sclera, encoding protein 1 of the low-density lipoprotein receptor family of cytokines and growth factors, and is involved in several cellular processes, even the negative regulation of gene expression [14]. *LRP1* deficiency leads to the disruption of TGF-β and may result in abnormal remodeling of the ECM of the developing eye, making this gene a candidate for HM [43].

Further studies are needed to demonstrate the direct involvement of these genes with EoHM for an accurate diagnosis, but for now, they are good candidates.

**Table 11:** Candidate genes of the OFT-00217 family.

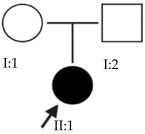	ACMG Criteria	ACMG Result	Variant	Zygosity	Inheritance	Total Families	Model of Inheritance	Gene Reported by
*AGRN*	PM2	VUS	NM_198576.3:c.4799C>T:p.(Ala1600Val)	Het	Paternal	2	AR	[40]
*CNTN6*	PM2, BP4	VUS	NM_014461.3:c.260A>G:p.(Asn87Ser)	Het	Paternal	1	Unknown	[42]
*CNTN6*	PM2, BP4	VUS	NM_014461.3:c.2553G>C:p.(Met851Ile)	Het	Paternal	1	Unknown	[42]
*FRMD4B*	PM2, PP3	VUS	NM_015123.2:c.554T>C:p.(Leu185Ser)	Het	Maternal	1	Unknown	[42]
*GRM6*	PM2, PVS1, PP5	LP	NM_000843.3:c.3G>T:p.(Met1?)	Het	Paternal	1	AR	[24,41]
*LRP1*	PM2, PP2	VUS	NM_002332.2:c.1415G>A:p.(Arg472Gln)	Het	Paternal	2	AD/AR	[43]

Het: heterozygous; AD: autosomal dominant; AR: autosomal recessive.

### 2.9. OFT-00223 Family

Candidate genes on Table 12. In the analysis of this family, we studied the alterations present in the two affected individuals. The protein encoded by *USH2A* is abundantly expressed in the macular and peripheral retina, mainly by photoreceptors. Alterations in this gene have been associated with Usher syndrome type IIa and retinitis pigmentosa. In a WES study of 20 patients with EoHM, *USH2A* was identified as a candidate gene, with pathogenic variants in four participants [19]. *ALKBH5* enables mRNA N6-methyladenosine dioxygenase activity, is involved in the response to hypoxia [15], and a decrease in its expression has been observed in individuals with EoHM [44].

This study proposes the involvement of both alterations in the development of EoHM.

### 2.10. OFT-00253 Family

Candidate genes on Table 13. In this family, we detected four genes that had not been observed in the other families studied. *CEP290* encodes the centrosomal protein 290, which is highly expressed in the retina of individuals with HM, being predominant in the retinal photoreceptors. Wan et al., proposed this gene as a candidate for causing EoHM [19].

*CPSF1* is known to play a role in mRNA processing; however, the relationship between *CPSF1* and human eye diseases, including myopia, remains unknown. Results from several studies suggest that mutations in *CPSF1* may be a new cause of EoHM [40,45,46]. *OPN4* encodes melanopsin, a photoreceptor opsin expressed in retinal ganglion and amacrine cells. These are necessary for correct refractive development and protection from myopia progression, as they are involved in the eye’s response to myopigenic stimuli, acting in part through dopaminergic mechanisms [47].

The proband was double heterozygous for the *MYOM1* gene, which is located at the MYP2 locus and is a structural constituent of the cytoskeleton thought to integrate the thin and thick filaments and confer elasticity to the M-band of the sarcomere in striated muscle. Alterations in this gene have been associated with the development of HM [48].

**Table 13:** Candidate genes of the OFT-00253 family.

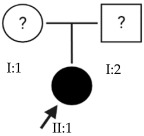	ACMG Criteria	ACMG Result	Variant	Zygosity	Inheritance	Total Families	Model of Inheritance	Gene Reported by
*CEP290*	PM2, BP4	VUS	NM_025114.3:c.6791A>G:p.(Lys2264Arg)	Het	Maternal	1	AR	[19]
*CPSF1*	PM2	VUS	NM_013291.2:c.3128C>T:p.(Pro1043Leu)	Het	Maternal	2	Unknown	[40,45,46]
*HSPG2*	PM2	VUS	NM_005529.6:c.3346G>A:p.(Gly1116Ser)	Het	Paternal	6	AD/AR	[19]
*MYOM1*	PM2, PP3	VUS	NM_003803.3:c.4580G>T:p.(Gly1527Val)	Het	Maternal	1	Unknown	[48]
*MYOM1*	PM2, PP3	VUS	NM_003803.3:c.3032T>C:p.(Val1011Ala)	Het	Paternal	1	Unknown	[48]
*OPN4*	BP6	VUS	NM_033282.3:c.1411_1412insT:p.(Ser473fs)	Het	Paternal	1	Unknown	[47]
*THBS2*	PM2	VUS	NM_003247.3:c.799G>A:p.(Glu267Lys)	Het	Paternal	2	Unknown	[28]

Het: heterozygous; AD: autosomal dominant; AR: autosomal recessive.

### 2.11. OFT-00268 Family

Candidate genes on Table 14. The proband, his mother (supposedly unaffected) and grandmother (affected) presented two alterations in the *CSMD1* gene that compromise its function and could be the cause of EoHM.

### 2.12. OFT-00332 Family

Candidate genes on Table 15. Most of the genes proposed in this family may have AD inheritance and affected individuals at different levels: retina (*ZNF644* and *ARHGEF15*), choroid (*CFH*) and sclera (*HSPG2* and *LRP1*), as well as *CPSF1*, which is known to be involved in the pathology although its exact cause is unknown. *ZNF644* encodes a zinc finger transcription factor in the RPE, regulating genes involved in ocular development. An alteration in this gene may therefore impact the structure of the eye, leading to the progression of EoHM. It has been related to the non-syndromic form of EoHM in several studies with an AD model of inheritance [15,49,50]. *ARHGEF15*, a guanine nucleotide exchange factor specific for RhoA, has been found to activate VEGF-induced Cdc42, promoting retinal angiogenesis [51].

*CFH* has antioxidant effects and regulates caspase-dependent apoptosis in retinal pigment epithelial cells under oxidative stress. This gene also blocks the pro-inflammatory effects of malondialdehyde, a major product of lipid peroxidation, and protects against oxidative stress in vivo in mice. Statistically significant higher levels of *CFH* have been observed in HM patients versus mild myopia and control groups, being greater in eyes with choroidal atrophy and its neovascularization, suggesting that it plays a key role in the development of myopia [52].

### 2.13. OFT-00403 Family

Candidate genes on Table 16. All three family members shared a single candidate variant, *TSG101*, which has a role in cell growth and differentiation and may act as a negative growth regulator. In the study by Le et al., the loss of *TSG101* severely altered the polarity of the RPE, forming irregular aggregates with a non-polarized distribution of cell adhesion proteins and the activation of epidermal growth factor receptor signaling [53]. In this particular case, the variant has a low allele frequency in the overall population and is synonymous; in an in silico splicing analysis using Alamut Visual 2.15 software, it was found unaltered by the variant. *TSG101* could therefore be a candidate for EoHM; however, further studies of this gene and its variants with other genes are necessary to determine the cause of the pathology.

**Table 16:** Candidate genes of the OFT-00403 family.

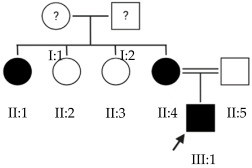	ACMG Criteria	ACMG Result	Variant	Zygosity	Inheritance	Total Families	Model of Inheritance	Gene Reported by
*TSG101*	PM2	VUS	NM_006292.3:c.942C>T:p.(Ile314Ile)	Het	Maternal	3	Unknown	[53]

Het: heterozygous.

### 2.14. OFT-00429 Family

Candidate genes on Table 17. The proband in this family had two deletions in the *HSPG2* gene found in previous families. As the alterations are close to each other, it may be observed that they are in cis, which means they were inherited from the same parent.

This individual also had an altered *LRPAP1* gene, which encodes a chaperone of LRP1, inhibits its degradation and influences the activity of TGF-β. An LRP1 deficiency and up-regulation of TGF-β have been observed in individuals with EoHM. TGF-β plays an important role in scleral ECM remodeling in myopia, and *LRPAP1* has been linked to EoHM in several studies [43,54].

*MMP9* is another gene involved in scleral ECM composition, implicated in the breakdown of type IV and V collagens [15,55]. Higher levels of *MMP9* have been found to reduce scleral elastin, making it more prone to deformation under intraocular pressure, linking it to the development of myopia and HM [55,56].

Finally, affecting the retina, we observed alterations in the *FLRT3* gene, which is involved in various signaling pathways and has been related to the development of the central nervous system and the eye due to its expression during ocular development in mouse embryos. It has also been associated with the development of HM in Central European families [57].

**Table 17:** Candidate genes of the OFT-00429 family.

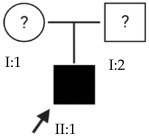	ACMG Criteria	ACMG Result	Variant	Zygosity	Inheritance	Total Families	Model of Inheritance	Gene Reported by
*FLRT3*	PM2, PP3	VUS	NM_013281.3:c.325T>G:p.(Leu109Val)	Het	Unknown	2	AD/Digenic/Multigenic	[57]
*HSPG2*	PM2, PM4	VUS	NM_005529.6:c.742_744delCTT:p.(Leu248del)	Het	Unknown	6	AD/AR	[19]
*HSPG2*	PVS1, PM2	LP	NM_005529.6:c.738delT:p.(Leu247fs)	Het	Unknown	6	AD/AR	[19]
*LRPAP1*	PM2	VUS	NM_002337.3:c.298G>A:p.(Gly100Ser)	Het	Unknown	1	AR	[43,54]
*MMP9*	PM2, BP4	VUS	NM_004994.2:c.1270C>A:p.(Arg424Ser)	Het	Unknown	1	AR	[55,56]

Het: heterozygous; AD: autosomal dominant; AR: autosomal recessive.

### 2.15. OFT-00474 Family

Candidate genes on Table 18. All the alterations selected as candidate genes in this proband were inherited from his father. The importance of *LAMA1* and *FRMPD1* in the development of EoHM has been discussed above. The only novel gene is *PRIMPOL*, a DNA primase-polymerase that facilitates DNA damage tolerance by mediating uninterrupted fork progression after UV irradiation and reinitiating DNA synthesis [15]. *PRIMPOL* mRNA, which is expressed in many tissues including scleral fibroblasts, retinal epithelial and Müller cells, has been associated with the development of HM in several studies [58,59,60].

### 2.16. OFT-00477 Family

Candidate genes on Table 19. The only candidate gene for the development of EoHM in this family was *TSG101*. This gene and its synonymous variant are the same as those observed in Family OFT-00403. Further study could be interesting to confirm or rule out its role in the pathology.

### 2.17. OFT-00506 Family

Candidate genes on Table 20. In this family, we identified a single candidate alteration in the *COL9A3* gene. This gene was also altered in family OFT-00155 and has an AD inheritance pattern, making it a good candidate.

### 2.18. OFT-00546 Family

Candidate genes on Table 21. The results for this family, like others in this study, suggest the cumulative effect of several altered genes involved in the development of EoHM inherited from both parents, although it includes genes that had not been observed in previous families. *ABCA4* is expressed in the outer segments of the cone and rod photoreceptors of the retina and in the sclera, mediating the transport of an essential molecule, all-trans-retinal aldehyde, across the photoreceptor cell membrane upon activation following phototransduction. Mutations in *ABCA4* can lead to multiple vision-related phenotypes, including retinitis pigmentosa, fundus flavimaculatus, cone-rod dystrophy and Stargardt disease. A variant in this gene responsible for myopia has recently been discovered, and has been proposed in the study by Wan et al., as a candidate gene for the development of EoHM [19].

*LAMA2* encodes the alpha-2 subunit of laminin, a major component of the basement membrane, with an important role in connecting the collagen fibers of the sclera. This gene has been linked to refractive error and myopia in different ethnic populations [61,62]. It may be that altered *LAMA2* and *LAMA5* have a greater effect on the development of EoHM than if they were isolated events, as observed in family OFT-00155t with *LAMA1* and *LAMA5*.

Alterations were also found in the *LTBP2* gene. This gene belongs to the family of latent TGF-β binding proteins, which are ECM proteins with a multidomain structure. It has been associated with Weill–Marchesani 3 and Stickler syndromes [15].

### 2.19. OFT-00586 Family

Candidate genes on Table 22. The alterations observed in this proband and her son affecting the development of EoHM primarily involve the retina. In addition to *LTBP2* and *TSG101*, which were also altered in previous families, this family presented altered *BICC1*. *BICC1*, located at the MYP15 locus [63], encodes an RNA-binding protein that regulates expression [15] and has been associated with the development of HM in several studies among different populations [63,64]. Alterations were also found in *CNTN4*, which encodes contactin-4, a glycosylphosphatidylinositol-anchored neuronal membrane protein that may play a role in the formation of axonal connections and arborization in the developing nervous system. *CNTN4* has been associated with HM in the literature [42].

### 2.20. OFT-00601 Family

Candidate genes on Table 23. Both affected members of this family had altered *CSMD1*, which is the candidate gene involved in the development of EoHM in this and other families included in our study.

### 2.21. OFT-00710 Family

Candidate genes on Table 24. In the last family, all the variants selected as candidates for the development of EoHM were maternally inherited. In addition to alterations in the *USH2A* and *LTBP2* genes, an alteration was also observed in *FBN1*, a member of the fibrillin family of proteins. Mutations in this gene are associated with Marfan syndrome (with HM as one of its clinical manifestations) and the MASS phenotype [15]. We propose all three genes as candidates for the progression of EoHM.

In our analysis of families with EoHM, we found that most of the alterations identified were classified as VUS, reflecting the need for further studies to determine the pathogenicity of these variants and to confirm the involvement of the proposed genes in the pathology. Table A2 shows a summary of the different variants found in each family, its classification, functional annotation and frequencies in the gnomAD global population. We also added the main pathways related to each gene.

The 74 VUS identified are located within a total of 47 genes. Out of these 47 genes, 19.15% are associated with pathologies that may manifest as HM without other clinical characteristics [37]. This percentage includes genes linked to myopia (*CPSF1*, *ZNF644*, *LRPAP1* and *PRIMPOL*, 8.51%), Stickler syndrome (*COL9A1*, *COL9A2* and *COL9A3*, 6.38%), Marfan syndrome (*FBN1*, 2.13%) and CSNB (*TRPM1*, 2.13%).

The remaining 80.85% of genes are associated with various other pathologies. However, this does not imply that they cannot contribute to EoHM, as previously discussed, since they play a role in the development of the eye and ocular structures and have been considered candidates in prior studies.

Five of the proposed genes codify transcription factors, which are *KDM6B*, *PER3*, *LRP1*, *ZNF644* and *PRIMPOL*.

**Figure 7:**
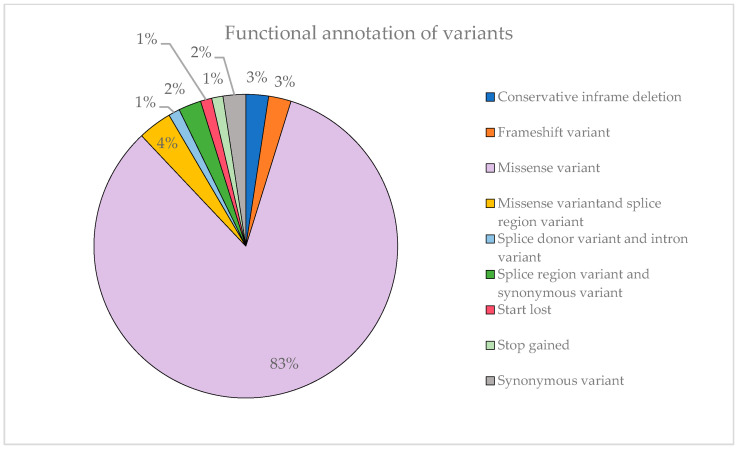
Representation of the functional annotation of variants and their proportion.

Figure 7 illustrates that most of the identified variants are missense mutations, accounting for 83% of them. This can explain the substantial proportion of variants classified as VUS observed previously. Missense variants can have a variable impact on the function of the protein encoded by the gene by altering a single amino acid and, moreover, not all options are covered. In contrast, stop-gained variants are far more likely to result in a complete loss of protein function, so they are rarely classified as VUS.

**Figure 8:**
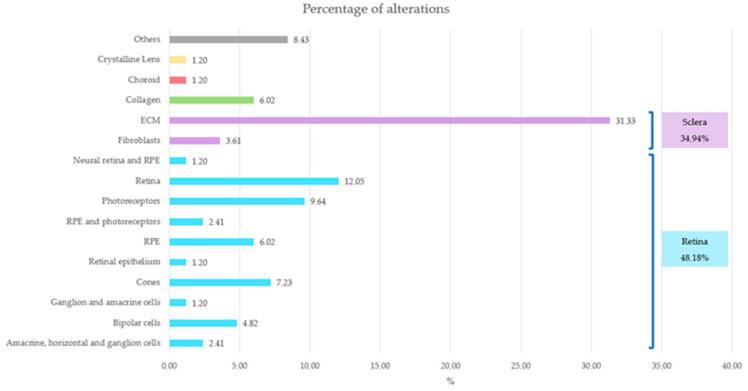
Representation of the affected ocular structures and their corresponding percentage. Retina structures are represented in blue, sclera structures in purple, choroid in red, crystalline lens in yellow, collagen in green and others in gray.

Notably, most alterations in the probands were primarily concentrated in the retina or sclera (Figure 8), an observation that coincides with the literature.

The main optical function of the crystalline lens is to transmit light, focusing it on the retina [65]. The retina is a layer of photoreceptor cells and glial cells that captures incoming photons from the lens, it can discern whether the perceived image is blurred or not, and it transmits the photons along neuronal pathways as both electrical and chemical signals for the brain to perceive a visual picture [66]. The choroid supplies the outer retina with nutrients and maintains the temperature and volume of the eye [67]. And the sclera influences eye size, facilitating the excessive axial elongation that occurs during myopigenesis [68]. If the variants impact genes that are expressed and have a function in ocular structures, their function may become compromised, leading to failures in processes such as visual acuity, light detection and regulation of ocular axial growth, resulting in a larger axial length in most of them. Among these, axial growth is particularly critical in the pathology under consideration.

Several genes were found to be altered in more than one family, suggesting a stronger association with the shared pathology. Genes with alterations in two or more families were *TRPM1*, *KDM6B*, *HSPG2*, *CACNA1F*, *CSMD1*, *LAMA1*, *COL9A3*, *LAMA5*, *THBS2*, *PCDH15*, *FRMPD1*, *AGRN*, *LRP1*, *USH2A*, *CPSF1*, *TSG101*, *FLRT3* and *LTBP2*. Specifically, *HSPG2* was altered in six of the twenty-one families, while *CSMD1* was altered in four, being the only candidate gene in several of them.

Although EoHM does not depend exclusively on the presence of alterations in these genes, it is influenced by the degree of penetrance, which was incomplete in some cases and unknown in others. Development of this pathology is also determined by the interaction and cumulative effect of these genes, with a genetic background affecting various levels of the multilayer signaling cascade (Figure 3 and Figure 8). Some genetic alterations may not have a significant impact in isolation in healthy individuals, but may have a more pronounced effect in the case of probands when combined with other alterations, thereby contributing to the development of the disease.

In addition to their biological function, most of the genes we propose have also been identified as candidates for EoHM in independent studies, supporting their involvement here.

## 3. Materials and Methods

A combined ophthalmological and genetic study was performed by the Multidisciplinary Unit of Ophthalmogenetics (UMOG) of the La Paz University Hospital, in accordance with the principles of the Declaration of Helsinki, and was approved by the ethics committee.

The inclusion criteria in our study were the following: (1) bilateral myopia with a refractive error of ≤−6 diopters in at least one eye with onset before the age of 10 years; (2) inconclusive result in the massive sequencing study implementing a panel (OFT-v3-1) of 419 genes related to ophthalmological disorders with suspected genetic cause, 93 genes and regions related to the pathogenesis of EoHM or within loci related to EoHM; (3) absence of syndromic phenotype; and (4) absence of corneal disease or other ophthalmologic diseases leading to secondary high myopia. In addition, whenever possible, parents and other relatives of the patients were included in the study.

**Figure 9:**
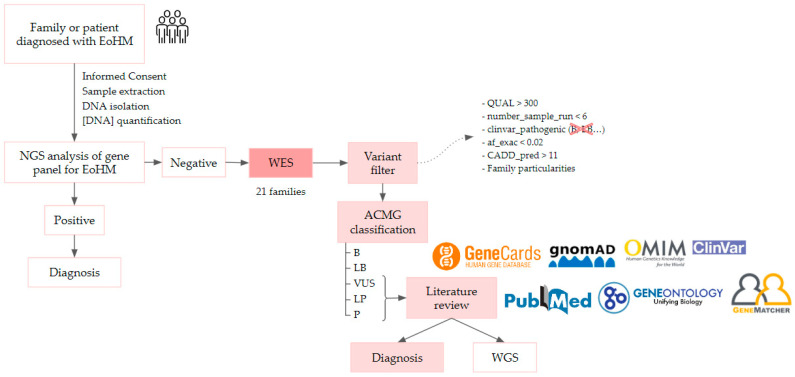
EoHM diagnostic workflow, it is described in more detail below. ACMG: American College of Medical Genetics; WES, whole-exome sequencing; WGS, whole-genome sequencing.

A total of 63 individuals from 21 unrelated families were recruited in accordance with the inclusion criteria, including 30 affected individuals aged between 6 and 80 years old. Once informed written consent was obtained from the probands and their parents or guardians, they entered the study and followed the workflow represented in Figure 9. The clinical ophthalmologic evaluation of patients and first-degree relatives who signed the consent form was performed by the Ophthalmology unit of Hospital La Paz (HULP-3576).

The method followed for the statistical analysis was a Fisher’s exact test to determine whether severity is dependent on gender, with the following hypotheses:

Null Hypothesis (H_0_): Severity and gender are independent variables.

Alternative Hypothesis (H_1_): Severity and gender are dependent variables.

The Fisher’s exact test was conducted using the ‘fisher.test()’ function in RStudio.

Participants first underwent a complete ophthalmological evaluation, including best-corrected visual acuity, refraction before and after cycloplegia, funduscopic examination, LA measurement, retinography and OCT imaging.

A genetic study was then performed on genomic DNA obtained from leukocytes isolated from a peripheral venous blood sample in the pre-analytical area of our institute using the Chemagic Magnetic Separation Module I (Chemagen, PerkinElmer, Waltham, MA, USA). Concentrations of the isolated DNA were quantified using a NanoDrop ND-1000 spectrophotometer (ThermoFisher Scientific, Waltham, MA, USA). Library preparation was carried out using Nextera DNA Exome (Illumina DNA Prep with Enrichment) and IDT for Illumina DNA/RNA UD Indexes Set A, B, C or D, Tagmentation. Sequencing was performed on high-quality sequencers, HiSeq4000 and NovaSeq6000, capturing 19,433 genes using xGen™ Exome Research Panel v2 IDT.

The Clinical Bioinformatics team at the Institute of Medical and Molecular Genetics (INGEMM) then performed the first analysis of the sequences obtained using an analytical algorithm developed to identify single nucleotide polymorphisms (SNPs) and insertions and deletions of small DNA fragments (indels) within the exome capture regions. This process comprises a sample pre-processing step, alignment of reads to a reference genome, identification and functional annotation of variants, and filtering of those variants. All these steps employ open tools widely used in the scientific community as well as proprietary tools. In addition, the steps are robustly designed and include process parameters and adequate quality controls to deliver a reliable report on the variants in question.

After sequencing, basecall file conversion (BCL) to FASTQ files was performed using Illumina’s demultiplexing software (bcl2fastq2 v2.20.0). The resulting DNA sequence reads were aligned and mapped to the human genome reference sequence (GRCh37/hg19) using bowtie2-align v2.0.6, after pre-processing and trimming using Trimmomatic v0.36 software. Realignment and recalibration of the reads were performed using the Genome Analysis Toolkit (GATK v3.3.0), and PCR duplicates were removed using Picard-tools v1.141. In addition, samtools v1.3.1 and BEDtools v2.26 software tools were used for the bioinformatics statistical analysis. SNPs and indels were detected using GATK v3.3.0, and the algorithms used for CNV detection were LACONv (unpublished in-house-developed algorithm) and eXome-Hidden Markov Model (XHMM v1.1). The variants were annotated in the Variant Call File (VCF) with predicted functional effect using SnpEff v4.3s. Furthermore, the following databases were also used for annotation: dbNSFP v3.5, dbSNP v151, ClinVar date 20180930, ExAC-1, SIFT ensembl 66, Polyphen-2 v2.2.2, MutationAssessor v3, FATHMM v2.3, CADD v1.4 and dbscSNV1.1.

The second analysis consisted of assessing the clinical significance of the variants found in the patients, relating them to their phenotype. To do so, we first filtered the data from bioinformatics using the criteria indicated in Table 25.

The variants were then classified according to ACMG prioritization standards with Franklin by Genoox, and doing so, we can know if the classification of the variant is B, LB, VUS, LP or P. Finally, we studied the clinical pathogenic significance of the VUS, LP and P variants and their relationship to the pathology under study; it was assessed by consulting several databases. The main databases consulted were Pubmed (pubmed.ncbi.nlm.nih.gov/), GeneCards (genecards.org/), gnomAD (gnomad.broadinstitute.org/), OMIM (omim.org/), Clinvar (ncbi.nlm.nih.gov/clinvar/) and GeneOntologyResource (geneontology.org/), as well as contacting different scientists through GeneMatcher (genematcher.org/).

The criteria followed to identify the candidate genes causing the pathology were scientific articles that (1) include the described gene involved in EoHM; (2) report the gene as a candidate implicated in EoHM, HM or syndromes that include EoHM among their manifestations; or (3) involve the development, homeostasis or correct functioning of the main tissues affected in EoHM (retina, choroid and sclera).

Candidate variants in affected subjects and relatives with indications of a causal relationship with EoHM should be validated using Sanger sequencing and functional studies. If no candidate variants are found, it would be necessary to extend the study with other techniques such as whole-genome sequencing (WGS).

## 4. Conclusions

Using WES, this study proposes 51 candidate genes that may cause EoHM. The genes identified are *TRPM1*, *ARHGEF18*, *KDM6B*, *HSPG2*, *COL9A2*, *FBLN1*, *CACNA1F*, *CSMD1*, *ADAMTSL1*, *LAMA1*, *COL9A3*, *LAMA5*, *THBS2*, *THBS1*, *PCDH15*, *BMPR2*, *LRP2*, *MAP3K1*, *LAMA4*, *PLG*, *VASH1*, *PER3*, *COL11A1*, *FRMPD1*, *GLB1*, *COL9A1*, *AGRN*, *GRM6*, *LRP1*, *CNTN6*, *FRMD4B*, *USH2A*, *ALKBH5*, *CEP290*, *CPSF1*, *OPN4*, *MYOM1*, *ZNF644*, *CFH*, *ARHGEF15*, *TSG101*, *LRPAP1*, *FLRT3*, *MMP9*, *PRIMPOL*, *ABCA4*, *LAMA2*, *LTBP2*, *BICC1*, *CNTN4* and *FBN1*.

The presence of several alterations associated with EoHM in the same patient may indicate the existence of incomplete penetrance or polygenic inheritance of the disease, suggesting a cumulative pathogenic effect of different VUSs. Further studies of the proposed candidate genes are needed to learn more about their actual involvement in EoHM and their cumulative effect. Such studies could include WES with different cohorts, WGS, transcriptome analysis, DNA methylation or long-read sequencing of these genes.

Sharing the results obtained in this study in conjunction with other previously published reports in the literature will contribute to a more accurate diagnosis of patients with EoHM in the future.

Our study also underscores the importance of the coordinated work of multidisciplinary teams to enhance patient care and diagnosis.

## Figures and Tables

**Table 1:** Clinical evaluation of probands.

Family ID	Gender	Actual Age	BCVA OD	BCVA OS	AL OD	AL OS	Funduscopic Examination OD	Funduscopic Examination OS	SPcc OD	Astig OD	SE OD cc	SPcc OS	Astig OS	SE OS cc
OFT-00074	F	16	0.6	0.08	26.6	26.93	Diffuse chorioretinal atrophy, central staphyloma	Diffuse chorioretinal atrophy, central staphyloma	−12	−0.5	−12.25	−12.75	−2.5	−14
OFT-00097	M	8	0.4	0.2	26.84	26.47	Tessellated fundus, healthy optic nerve	Tessellated fundus, healthy optic nerve	−9.75	−5.25	−12.35	−10	−5.25	−12.6
OFT-00155	M	12	0.125	0.1	NA	NA	Healthy retina	Healthy retina	−10	−1.25	−10.6	−8.75	−2.75	−10.1
OFT-00175	M	16	0.9	0.8	31.22	30.98	Diffuse chorioretinal atrophy, peripapillary atrophy	Diffuse chorioretinal atrophy, peripapillary atrophy	−13.5	−4.75	−15.87	−13.25	−6	−16.25
OFT-00178	M	7	0.3	0.4	26.75	26.65	Healthy retina, mild optic nerve pallor	Healthy retina	−6.75	−4	−8.75	−7.25	−3.25	−8.88
OFT-00191	M	8	0.5	0.5	26.05	26.15	Diffuse chorioretinal atrophy, mild optic nerve pallor	Diffuse chorioretinal atrophy, mild optic nerve pallor	−9	−2	−10	−8.75	−3.25	−10.4
OFT-00209	M	10	0.6	0.7	NA	NA	Diffuse chorioretinal atrophy	Diffuse chorioretinal atrophy	−8.5	−3	−10	−7	−3	−8.5
OFT-00217	F	11	0.8	0.8	NC	NC	Normal	Normal	−7.00	−1.75	−7.75	−8.75	−1.50	−9.50
OFT-00223	F	9	0.3	0.3	28.04	27.62	Atrophic optic nerve	Atrophic optic nerve	−13.5	−2.5	−14.75	−13	−0.5	−13.25
OFT-00253	F	7	0.9	0.9	29.59	29.1	Healthy retina	Healthy retina	−19.25	0	−19.25	−17.25	−0.5	−17.5
OFT-00268	M	9	0.5	0.6	27.08	27.18	Diffuse chorioretinal atrophy	Diffuse chorioretinal atrophy	−7.25	−0.75	−7.6	−7	−1	−7.5
OFT-00332	M	12	0.25	0.3	29.41	29.02	Tessellated fundus, epiretinal fibrosis	Tessellated fundus, WWP on inferior and temporal retina	−15.25	−1	−15.75	−14.75	−0.5	−15
OFT-00403	M	9	1	0.8	NC	NC	Normal	Normal	−14.75	−1.00	−15.25	−15.00	−0.75	−15.25
OFT-00429	M	36	0.8	0.6	NA	NA	Diffuse chorioretinal atrophy, peripapillary atrophy, WWP inferiorly	Diffuse chorioretinal atrophy, peripapillary atrophy, WWP inferiorly	−20	0	−20	−19	0	−19
OFT-00474	M	6	0.1	0.7	27.43	25.99	Diffuse chorioretinal atrophy	Diffuse chorioretinal atrophy	−11.5	−1.25	−12.125	−10.25	−0.5	−10.5
OFT-00477	F	11	1.25	1.25	NA	NA	Healthy retina	Healthy retina	−7.5	−0.75	−7.875	−8	−1.5	−8.75
OFT-00506	F	16	0.7	0.7	NA	NA	Tessellated fundus	Tessellated fundus	−13.25	−2	−14.25	−12.5	−1.5	−13.25
OFT-00546	M	12	1	1	24.45	24.12	Healthy retina	Healthy retina	−7.25	−0.75	−7.625	−5	−1	−5.5
OFT-00586	F	80	0.8	0.05	NA	NA	Diffuse chorioretinal atrophy	Diffuse chorioretinal atrophy	NA	NA	NA	NA	NA	NA
OFT-00601	M	13	1	0.9	27.92	27.68	Healthy retina	Healthy retina	−7	−1	−7.5	−6.5	−1	−7
OFT-00710	M	10	0.8	0.9	NC	NC	Peripapillary atrophy	Peripapillary atrophy	−8.50	−5.50	−10.25	−6.00	−6.00	−9.00

M, male; F, female; BCVA, best-corrected visual acuity; NFx, non-fixation; Fx, fixation; NLP, no light perception; OD, right eye; OS, left eye; AL, axial length; WWP, white without pressure; RD, retinal detachment; SPcc, sphere with cycloplegia; Astig, astigmatism; SE, spherical equivalent; NA, not available.

**Table 2:** Refractive results.

	Right Eye	Left Eye
SP cc (diopters)	−11.21 ± 4.098	−10.632 ± 4.034
Astig (diopters)	−1.95 ± 1.697	−2.113 ± 1.858
SE (diopters)	−12.044 ± 3.894	−11.499 ± 3.81
BCVA (decimal scale)	0.649 ± 0.319	0.599 ± 0.333

Results are presented as mean ± standard deviation.

**Table 3:** Frequencies.

	Female	Male	Total Female and Male
**More severe (<−10 SE cc)**	5	8	13
**Less severe (>−10 SE cc)**	2	6	8
**Total More and Less severe**	7	14	21

**Table 6:** Candidate genes of the OFT-00155 family.

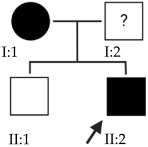	ACMG Criteria	ACMG Result	Variant	Zygosity	Inheritance	Total Families	Model of Inheritance	Gene Reported by
*CACNA1F*	PP3, PM2	LP	NM_005183.3:c.2924G>A:p.(Arg975Gln)	Hemi	Maternal *	2	XL	[22,24]
*COL9A3*	-	VUS	NM_001853.3:c.1258C>G:p.(Gln420Glu)	Het	Maternal	2	AD	[15]
*HSPG2*	PM2	VUS	NM_005529.6:c.4493C>T:p.(Ser1498Phe)	Het	Maternal *	6	AD/AR	[19]
*LAMA1*	PM2, BP4	VUS	NM_005559.3:c.3958A>G:p.(Ile1320Val)	Het	Maternal *	2	AR	[25,26,27]
*LAMA5*	PM2	VUS	NM_005560.4:c.1744C>T:p.(Pro582Ser)	Het	Maternal *	2	Unknown	[15]
*THBS1*	PM2, BP4	VUS	NM_003246.3:c.1122C>T:p.(Pro374Pro)	Het	Maternal *	1	Unknown	[28]
*THBS2*	PM2	VUS	NM_003247.3:c.1019C>T:p.(Thr340Met)	Het	Maternal*	2	Unknown	[28]

Het: heterozygous; Hemi: hemizygous; AD: autosomal dominant; AR: autosomal recessive; XL: X-linked. * Alterations shared with his brother (he has idiopathic motor nystagmus).

**Table 10:** Candidate genes of the OFT-00209 family.

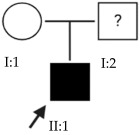	ACMG Criteria	ACMG Result	Variant	Zygosity	Inheritance	Total Families	Model of Inheritance	Reported Gene by
*COL9A1*	PM2, BP1	VUS	NM_001851.4:c.6G>T:p.(Lys2Asn)	Het	Unknown	1	AR	[15]
*GLB1*	PM2, PM5, PP3, PP2, PP5	P	NM_000404.3:c.1498A>G:p.(Thr500Ala)	Het	Unknown	1	AR	[39]
*KDM6B*	PM2, BP4	VUS	NM_001080424.1:c.3221C>G:p.(Ala1074Gly)	Het	Unknown	2	Unknown	[17]

Het: heterozygous; AR: autosomal recessive.

**Table 12:** Candidate genes of the OFT-00223 family.

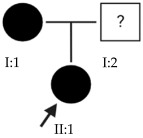	ACMG Criteria	ACMG Result	Variant	Zygosity	Inheritance	Total Families	Model of Inheritance	Gene Reported by
*ALKBH5*	PM2, BP4	VUS	NM_017758.3:c.952C>A:p.(Pro318Thr)	Het	Maternal	1	Unknown	[44]
*USH2A*	PM2, BP4	VUS	NM_206933.2:c.15172T>C:p.(Phe5058Leu)	Het	Maternal	2	AR	[19,24]

Het: heterozygous; AR: autosomal recessive.

**Table 14:** Candidate gene of the OFT-00268 family.

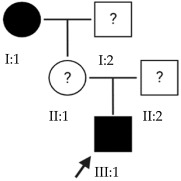	ACMG Criteria	ACMG Result	Variant	Zygosity	Inheritance	Total Families	Model of Inheritance	Gene Reported by
*CSMD1*	PM2, PP3	VUS	NM_033225.5:c.9089C>G:p.(Pro3030Arg)	Het	Maternal	4	Unknown	[19,23]
*CSMD1*	PM2, BP4	VUS	NM_033225.5:c.7050T>A:p.(Ser2350Arg)	Het	Maternal	4	Unknown	[19,23]

Het: Heterozygous.

**Table 15:** Candidate genes of the OFT-00332 family.

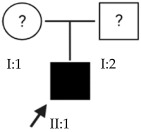	ACMG Criteria	ACMG Result	Variant	Zygosity	Inheritance	Total Families	Model of Inheritance	Gene Reported by
*ARHGEF15*	PM2, BP4	VUS	NM_173728.3:c.380C>G:p.(Pro127Arg)	Het	Paternal	1	Unknown	[51]
*CFH*	PM2, BS2	VUS	NM_000186.3:c.481G>T:p.(Ala161Ser)	Het	Maternal	1	AD/AR	[52]
*CPSF1*	PM2, PP3	VUS	NM_013291.2:c.2383G>A:p.(Glu795Lys)	Het	Paternal	2	Unknown	[40,45,46]
*HSPG2*	PM2	VUS	NM_005529.6:c.4078A>G:p.(Asn1360Asp)	Het	Paternal	6	AD/AR	[19]
*LRP1*	PM2, PP2, BP6	VUS	NM_002332.2:c.11930C>T:p.(Ser3977Leu)	Het	Paternal	2	AD/AR	[43]
*ZNF644*	PM2	VUS	NM_201269.2:c.1366A>T:p.(Thr456Ser)	Het	Maternal	1	AD	[49,50]

Het: heterozygous; AD: autosomal dominant; AR: autosomal recessive.

**Table 18:** Candidate genes of the OFT-00474 family.

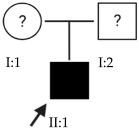	ACMG Criteria	ACMG Result	Variant	Zygosity	Inheritance	Total Families	Model of Inheritance	Gene Reported by
*FRMPD1*	PM2, PP3	VUS	NM_014907.2:c.925G>A:p.(Ala309Thr)	Het	Paternal	2	Unknown	[38]
*LAMA1*	-	VUS	NM_005559.3:c.781A>G:p.(Ile261Val)	Het	Paternal	2	AR	[25,26,27]
*PRIMPOL*	PM2, BP4	VUS	NM_152683.3:c.1380T>C:p.(Cys460Cys)	Het	Paternal	1	AD	[58,59,60]

Het: heterozygous; AD: autosomal dominant; AR: autosomal recessive.

**Table 19:** Candidate gene of the OFT-00477 family.

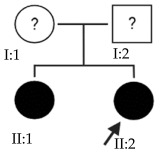	ACMG Criteria	ACMG Result	Variant	Zygosity	Inheritance	Total Families	Model of Inheritance	Gene Reported by
*TSG101*	PM2	VUS	NM_006292.3:c.942C>T:p.(Ile314Ile)	Het	Paternal	3	Unknown	[53]

Het: heterozygous.

**Table 20:** Candidate gene of the OFT-00506 family.

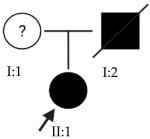	ACMG Criteria	ACMG Result	Variant	Zygosity	Inheritance	Total Families	Model of Inheritance	Gene Reported by
*COL9A3*	PM2	VUS	NM_001853.3:c.1511C>T:p.(Pro504Leu)	Het	Unknown	2	AD	[15]

Het: heterozygous.

**Table 21:** Candidate genes of the OFT-00546 family.

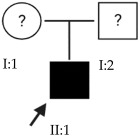	ACMG Criteria	ACMG Result	Variant	Zygosity	Inheritance	Total Families	Model of Inheritance	Gene Reported by
*ABCA4*	PS4, PP3, PP2, BS1, PP5, BP6	VUS	NM_000350.2:c.6148G>C:p.(Val2050Leu)	Het	Paternal	1	AR	[19,24]
*AGRN*	PM2	VUS	NM_198576.3:c.2737G>A:p.(Val913Met)	Het	Paternal	2	AR	[40]
*FLRT3*	PM2	VUS	NM_013281.3:c.1135G>A:p.(Gly379Arg)	Het	Maternal	2	AD/Digenic/Multigenic	[57]
*LAMA2*	PM2	VUS	NM_000426.3:c.6880G>T:p.(Val2294Leu)	Het	Paternal	1	AD/AR	[61,62]
*LAMA5*	PM2	VUS	NM_005560.4:c.11063G>A:p.(Gly3688Glu)	Het	Maternal	2	Unknown	[15]
*LTBP2*	PM2, PP3	VUS	NM_000428.2:c.3998G>C:p.(Gly1333Ala)	Het	Paternal	3	AR	[15,24]

Het: heterozygous; AD: autosomal dominant; AR: autosomal recessive.

**Table 22:** Candidate genes of the OFT-00586 family.

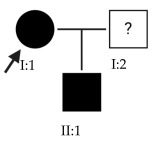	ACMG Criteria	ACMG Result	Variant	Zygosity	Inheritance	Total Families	Model of Inheritance	Gene Reported by
*BICC1*	PM2	VUS	NM_001080512.2:c.1425G>C:p.(Leu475Phe)	Het	Maternal	1	AD	[63,64]
*CNTN4*	PM2	VUS	NM_001206955.1:c.2128G>A:p.(Gly710Arg)	Het	Maternal	1	Unknown	[42]
*LTBP2*	BP4	VUS	NM_000428.2:c.1487G>A:p.(Gly496Asp)	Het	Maternal	3	AR	[15,24]
*TSG101*	PM2	VUS	NM_006292.3:c.307G>A:p.(Val103Ile)	Het	Maternal	3	Unknown	[53]

Het: heterozygous; AD: autosomal dominant; AR: autosomal recessive.

**Table 23:** Candidate gene of the OFT-00601 family.

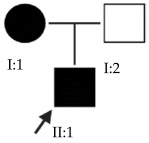	ACMG Criteria	ACMG Result	Variant	Zygosity	Inheritance	Total Families	Model of Inheritance	Gene Reported by
*CSMD1*	PM2	VUS	NM_033225.5:c.5600C>G:p.(Pro1867Arg)	Het	Maternal	4	Unknown	[19,23]

Het: heterozygous.

**Table 24:** Candidate genes of the OFT-00710 family.

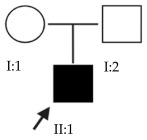	ACMG Criteria	ACMG Result	Variant	Zygosity	Inheritance	Total Families	Model of Inheritance	Gene Reported by
*FBN1*	PM2, PP2	VUS	NM_000138.4:c.8195G>C:p.(Ser2732Thr)	Het	Maternal	1	AD	[15]
*LTBP2*	PM2	VUS	NM_000428.2:c.2512G>A:p.(Val838Met)	Het	Maternal	3	AR	[15,24]
*USH2A*	PM2, BP4	VUS	NM_206933.2:c.9776T>C:p.(Val3259Ala)	Het	Maternal	2	AR	[19,24]

Het: heterozygous; AD: autosomal dominant; AR: autosomal recessive.

**Table 25:** Filters used after the bioinformatic analysis.

Code	Description	Filter
QUAL	Quality of the variation found.	>300
number_sample_run	Number of people in that run with the same variant.	<6
clinvar_pathogenic	Prediction of variant pathogenicity based on Clinvar database.	Benign and Likely Benign discarded
af_exac_all	Allele frequency in the global population according to Exome Aggregation Consortium (ExAC).	<0.02
af_exac_afr	Allele frequency in the African population according to ExAC.	<0.02
af_exac_amr	Allele frequency in the American population according to ExAC.	<0.02
af_exac_eas	Allele frequency in the East Asian population according to ExAC.	<0.02
af_exac_fin	Allele frequency in the Finnish population according to ExAC.	<0.02
af_exac_nfe	Allele frequency in the Non-Finnish European population according to ExAC.	<0.02
af_exac_sas	Allele frequency in the South Asian population according to ExAC.	<0.02
af_exac_oth	Allele frequency in the other population according to ExAC.	<0.02
CADD_pred	Prediction of variant pathogenicity based on Combined Annotation Dependent Depletion (CADD) score.	Benign discarded
Family particularities	Depending on the affected subjects in every family.	

## Data Availability

Not applicable.

## Abbreviations

ACMG	American College of Medical Genetics
AD	Autosomal dominant
ADAMTS	A disintegrin and metalloproteinase with thrombospondin motif
AL	Axial length
AR	Autosomal recessive
Astig	Astigmatism
BCVA	Best-corrected visual acuity
BMP	Bone morphogenetic protein
CSNB	Congenital stationary night blindness
ECM	Extracellular matrix
EDP	Elastin-derived peptides
EoHM	Early-onset High Myopia
Fx	Fixation
GEF	Guanine nucleotide exchange factor
Gpsm2	G-protein signaling modulator 2
GWAS	Genome-wide association study
Hemi	Hemizygous
Het	Heterozygous
HM	High myopia
LP	Likely pathogenic
NA	Not available
NFx	Non-fixation
NGS	Next-Generation Sequencing
NLP	No light perception
OD	Right eye
OS	Left eye
P	Pathogenic
RD	Retinal detachment
RPE	Retinal pigment epithelium
SD	Spherical diopters
SE	Spherical equivalent
SPcc	Sphere with cycloplegia
UMOG	Multidisciplinary Unit of Ophthalmogenetics
VUS	Variant of uncertain significance
WES	Whole-exome sequencing
WGS	Whole-genome sequencing
WWP	White without pressure
XL	X-linked

## Appendix A

**Table A1:** American College of Medical Genetics and Genomics criteria for classifying pathogenic variants.

**Code**	**Description**
**PVS1**	Null variant (nonsense, frameshift, canonical ±1 or 2 splice sites, initiation codon, single or multiexon deletion) in a gene where loss of function is a known mechanism of disease.
**PS1**	Same amino acid change as a previously established pathogenic variant regardless of nucleotide change.
**PS2**	De novo (both maternity and paternity confirmed) in a patient with the disease and no family history.
**PS3**	Well-established in vitro or in vivo functional studies supporting a damaging effect on the gene or gene product.
**BS1**	Allele frequency is greater than expected for the disorder.
**PM1**	Located in a mutational hotspot and/or critical and well-established functional domain (e.g., active site of an enzyme) with no benign variation.
**PM2**	Absent from controls (or at extremely low frequency if recessive) in Exome Sequencing Project, 1000 Genomes Project, and Exome Aggregation Consortium.
**PM3**	For recessive disorders, detected in trans with a pathogenic variant.
**PM4**	Protein length changes as a result of in-frame deletions/insertions in a non-repeat region or stop-loss variants.
**PM5**	Novel missense change at an amino acid residue where a different missense change determined to be pathogenic has been previously observed.
**PP1**	Cosegregation with disease in multiple affected family members in a gene definitively known to cause the disease.
**PP2**	Missense variant in a gene that has a low rate of benign missense variation and in which missense variants are a common mechanism of disease.
**PP3**	Multiple lines of computational evidence support a deleterious effect on the gene or gene product (conservation, evolutionary, splicing impact, etc.)
**PP5**	Reputable source recently reports variant as pathogenic, but the evidence is not available to the laboratory to perform an independent evaluation.
**BP4**	Multiple lines of computational evidence suggest no impact on gene or gene product (conservation, evolutionary, splicing impact, etc.)
**BP6**	Reputable source recently reports variant as benign, but the evidence is not available to the laboratory to perform an independent evaluation.

**Table A2:** Summary of the different variants found in each family.

**Family**	**Variant**	**Classification**	**Functional Annotation**	**GnomAD Allele Frequency**	**Gene**	**Gene Ontology Main Pathways Associated**
**OFT-00074**	NM_015318.3:c.2167C>T:p.(Arg723Cys)	VUS	missense_variant	0	*ARHGEF18*	Small-GTPase-mediated signal transduction, negative regulation of stress fiber assembly, extracellular exosome in part of neural stem cell, regulation of Rho protein signal transduction, plasma membrane
	NM_001080424.1:c.1582C>T:p.(Pro528Ser)	VUS	missense_variant	0.00001577	*KDM6B*	Beta-catenin binding, histone demethylase activity, metal ion binding, histone h3k27me2/h3k27me3 demethylase activity, inflammatory response to antigenic stimulus, chromatin remodeling, positive regulation of transcription by RNA polymerase II, regulation of gene expression
	NM_002420.5:c.1023+1G>A	P	splice_donor_variant&intron_variant	0.00002005	*TRPM1*	Calcium channel activity, G-protein-coupled glutamate receptor signaling pathway, visual perception, cellular response to light stimulus, axon
**OFT-00097**	NM_001040272.5:c.1819G>A:p.(Glu607Lys)	VUS	missense_variant	0.0001279	*ADAMTSL1*	Hydrolase activity, extracellular matrix organization, extracellular region
	NM_005183.3:c.4504C>T:p.(Arg1502*)	P	stop_gained	0	*CACNA1F*	Voltage-gated calcium channel activity, metal ion binding, visual perception, detection of light stimulus involved in visual perception, photoreceptor outer segment
	NM_001852.3:c.1652C>T:p.(Ala551Val)	VUS	missense_variant	0.00005996	*COL9A2*	Extracellular matrix structural constituent conferring tensile strength, protein homodimerization activity, skeletal system development, extracellular region, endoplasmic reticulum lumen, collagen-containing extracellular matrix, collagen type IX trimer, extracellular space, extracellular matrix organization,
	NM_033225.5:c.1712A>G:p.(Asn571Ser)	VUS	missense_variant	0.00001450	*CSMD1*	Male gonad development, female gonad development, oviduct epithelium development
	NM_006486.2:c.1157C>T:p.(Thr386Met)	VUS	missense_variant	0.0001520	*FBLN1*	Collagen-containing extracellular matrix, fibronectin binding, integrin binding, extracellular matrix structural constituent, calcium ion binding, peptidase activator activity, fibrinogen binding, extracellular matrix organization, extracellular region, extracellular space, extracellular matrix, extracellular exosome
	NM_005529.6:c.12691G>A:p.(Glu4231Lys)	VUS	missense_variant	0.00003196	*HSPG2*	Collagen-containing extracellular matrix, amyloid-beta binding, calcium ion binding, extracellular matrix structural constituent conferring compression resistance, collagen V binding, angiogenesis, response to hypoxia, positive regulation of endothelial cell proliferation, inflammatory response, negative regulation of angiogenesis, extracellular region, extracellular space, extracellular exosome
**OFT-00155**	NM_005183.3:c.2924G>A:p.(Arg975Gln)	LP	missense_variant	0	*CACNA1F*	Voltage-gated calcium channel activity, metal ion binding, visual perception, detection of light stimulus involved in visual perception, photoreceptor outer segment
	NM_001853.3:c.1258C>G:p.(Gln420Glu)	VUS	missense_variant	0.0002642	*COL9A3*	Extracellular matrix structural constituent conferring tensile strength, protein homodimerization activity, extracellular region, collagen-containing extracellular matrix, collagen type IX trimer, extracellular matrix organization, extracellular space
	NM_005529.6:c.4493C>T:p.(Ser1498Phe)	VUS	missense_variant	0.00004378	*HSPG2*	Collagen-containing extracellular matrix, amyloid-beta binding, calcium ion binding, extracellular matrix structural constituent conferring compression resistance, collagen V binding, angiogenesis, response to hypoxia, positive regulation of endothelial cell proliferation, inflammatory response, negative regulation of angiogenesis, extracellular region, extracellular space, extracellular exosome
	NM_005559.3:c.3958A>G:p.(Ile1320Val)	VUS	missense_variant	0.000003977	*LAMA1*	Collagen-containing extracellular matrix, extracellular matrix structural constituent, glycosphingolipid binding, morphogenesis of an epithelial sheet, neuron projection development, establishment of epithelial cell apical/basal polarity, retinal blood vessel morphogenesis, positive regulation of integrin-mediated signaling pathway, extracellular region, extracellular space, extracellular matrix, collagen-containing extracellular matrix, protein complex involved in cell–matrix adhesion, laminin-1 complex, laminin-3 complex
	NM_005560.4:c.1744C>T:p.(Pro582Ser)	VUS	missense_variant	0.000004338	*LAMA5*	Collagen-containing extracellular matrix, integrin binding, extracellular matrix structural constituent, morphogenesis of a polarized epithelium, integrin-mediated signaling pathway, morphogenesis of embryonic epithelium, regulation of epithelial cell proliferation, postsynapse organization, extracellular region, synaptic cleft, collagen-containing extracellular matrix, extracellular exosome, extracellular matrix of synaptic cleft, laminin-5 complex, laminin-10 complex, laminin-11 complex
	NM_003246.3:c.1122C>T:p.(Pro374Pro)	VUS	splice_region_variant&synonymous_variant	0.00006059	*THBS1*	Positive regulation of MAP kinase activity, negative regulation of endothelial cell proliferation, negative regulation of cell–matrix adhesion, negative regulation of angiogenesis, collagen-containing extracellular matrix, fibronectin binding, integrin binding, extracellular matrix structural constituent, calcium ion binding, fibroblast growth factor binding, laminin binding, proteoglycan binding, transforming growth factor beta binding, fibrinogen binding, collagen V binding, response to hypoxia, sprouting angiogenesis, chronic inflammatory response, apoptotic process, inflammatory response, positive regulation of fibroblast migration, negative regulation of fibroblast growth factor receptor signaling pathway, positive regulation of extrinsic apoptotic signaling pathway via death domain receptors, positive regulation of reactive oxygen species metabolic process, extracellular region, extracellular space, extracellular matrix, fibrinogen complex
	NM_003247.3:c.1019C>T:p.(Thr340Met)	VUS	missense_variant	0.00008847	*THBS2*	Extracellular matrix structural constituent, calcium ion binding, protein binding, negative regulation of angiogenesis, positive regulation of synapse assembly, extracellular region, collagen-containing extracellular matrix
**OFT-00175**	NM_001204.6:c.1931A>G:p.(Asn644Ser)	VUS	missense_variant	0	*BMPR2*	Positive regulation of gene expression, positive regulation of SMAD protein signal transduction, positive regulation of epithelial cell migration, proteoglycan biosynthetic process, positive regulation of transcription by RNA polymerase II, retina vasculature development in camera-type eye, cellular response to BMP stimulus, endothelial cell apoptotic process, extracellular space, nucleoplasm, plasma membrane, transforming growth factor beta receptor activity
	NM_005529.6:c.10481G>A:p.(Arg3494Gln)	VUS	missense_variant	0.00001199	*HSPG2*	Collagen-containing extracellular matrix, amyloid-beta binding, calcium ion binding, extracellular matrix structural constituent conferring compression resistance, collagen V binding, angiogenesis, response to hypoxia, positive regulation of endothelial cell proliferation, inflammatory response, negative regulation of angiogenesis, extracellular region, extracellular space, extracellular exosome
	NM_001142769.1:c.4396A>G:p.(Ser1466Gly)	VUS	missense_variant	0.000004036	*PCDH15*	Sensory perception of sound, calcium ion binding, photoreceptor cell maintenance, sensory perception of light stimulus, photoreceptor outer segment, extracellular region
	NM_002420.5:c.4433C>T:p.(Thr1478Met)	VUS	missense_variant	0.0005196	*TRPM1*	Calcium channel activity, G-protein-coupled glutamate receptor signaling pathway, visual perception, cellular response to light stimulus, axon
**OFT-00178**	NM_033225.5:c.8042G>A:p.(Gly2681Asp)	VUS	missense_variant	0.0003290	*CSMD1*	Male gonad development, female gonad development, oviduct epithelium development
	NM_033225.5:c.4375G>A:p.(Ala1459Thr)	VUS	missense_variant	0.00006418	*CSMD1*	Male gonad development, female gonad development, oviduct epithelium development
	NM_001105207.2:c.673G>A:p.(Ala225Thr)	VUS	missense_variant	0.00001994	*LAMA4*	Collagen-containing extracellular matrix, extracellular matrix structural constituent, extracellular region
	NM_004525.2:c.10202C>G:p.(Thr3401Arg)	VUS	missense_variant	0.00004599	*LRP2*	Negative regulation of apoptotic process, vitamin D metabolic process, phosphatidylinositol 3-kinase/protein kinase B signal transduction, cellular response to growth factor stimulus, neuron projection arborization, extracellular exosome
	NM_005921.1:c.299G>A:p.(Gly100Glu)	VUS	missense_variant	0	*MAP3K1*	Protein serine/threonine kinase activity, MAP kinase activity, MAPK cascade, protein phosphorylation, Fc-epsilon receptor signaling pathway
	NM_005921.1:c.3646_3648delATC:p.(Ile1216del)	VUS	conservative_inframe_deletion	0.000008190	*MAP3K1*	Protein serine/threonine kinase activity, MAP kinase activity, MAPK cascade, protein phosphorylation, Fc-epsilon receptor signaling pathway
	NM_001142769.1:c.1519G>A:p.(Val507Ile)	VUS	missense_variant	0.00009902	*PCDH15*	Sensory perception of sound, calcium ion binding, photoreceptor cell maintenance, sensory perception of light stimulus, photoreceptor outer segment, extracellular region
	NM_000301.3:c.598A>G:p.(Thr200Ala)	VUS	missense_variant	0.0007181	*PLG*	Endopeptidase activity, serine-type endopeptidase activity, extracellular matrix disassembly, modulating synaptic transmission, negative regulation of cell–cell adhesion mediated by cadherin, extracellular region, extracellular space, collagen-containing extracellular matrix, extracellular exosome
	NM_014909.4:c.953G>A:p.(Arg318Gln)	VUS	missense_variant	0	*VASH1*	Angiogenesis, negative regulation of endothelial cell proliferation, negative regulation of angiogenesis, extracellular space
**OFT-00191**	NM_001854.3:c.2900G>T:p.(Gly967Val)	LP	missense_variant	0	*COL11A1*	Extracellular matrix structural constituent, extracellular matrix structural constituent conferring tensile strength, extracellular matrix binding, proteoglycan metabolic process, visual perception, extracellular matrix organization, collagen fibril organization, collagen type XI trimer, endoplasmic reticulum lumen, collagen-containing extracellular matrix, collagen type XI trime
	NM_014907.2:c.2469C>A:p.(Ser823Arg)	VUS	missense_variant	0.0002050	*FRMPD1*	Regulation of G-protein-coupled receptor signaling pathway
	NM_016831.2:c.3502A>G:p.(Thr1168Ala)	VUS	missense_variant	0.0005746	*PER3*	Negative regulation of transcription by RNA polymerase II, regulation of circadian sleep/wake cycle, sleep, protein stabilization, circadian regulation of gene expression, transcription cis-regulatory region binding
**OFT-00209**	NM_001851.4:c.6G>T:p.(Lys2Asn)	VUS	missense_variant	0.00004374	*COL9A1*	Extracellular matrix structural constituent conferring tensile strength, extracellular region, collagen-containing extracellular matrix, collagen type IX trimer, extracellular matrix organization, extracellular space
	NM_000404.3:c.1498A>G:p.(Thr500Ala)	P	missense_variant	0.00001216	*GLB1*	Beta-galactosidase activity, glycosphingolipid metabolic process, galactose catabolic process, heparan sulfate proteoglycan catabolic process
	NM_001080424.1:c.3221C>G:p.(Ala1074Gly)	VUS	missense_variant	0	*KDM6B*	Beta-catenin binding, histone demethylase activity, metal ion binding, histone h3k27me2/h3k27me3 demethylase activity, inflammatory response to antigenic stimulus, chromatin remodeling, positive regulation of transcription by RNA polymerase II, regulation of gene expression
**OFT-00217**	NM_198576.3:c.4799C>T:p.(Ala1600Val)	VUS	missense_variant	0.00005405	*AGRN*	Collagen-containing extracellular matrix, extracellular matrix structural constituent, laminin binding, heparan sulfate proteoglycan binding, clustering of voltage-gated sodium channels, positive regulation of transcription by RNA polymerase II, extracellular region, collagen-containing extracellular matrix, extracellular exosome, neuromuscular junction development
	NM_014461.3:c.260A>G:p.(Asn87Ser)	VUS	missense_variant	0.00002830	*CNTN6*	Notch signaling pathway, central nervous system development, axon guidance
	NM_014461.3:c.2553G>C:p.(Met851Ile)	VUS	missense_variant	0.00001194	*CNTN6*	Notch signaling pathway, central nervous system development, axon guidance
	NM_015123.2:c.554T>C:p.(Leu185Ser)	VUS	missense_variant	0.00005346	*FRMD4B*	Establishment of epithelial cell polarity, ruffle, extracellular space
	NM_000843.3:c.3G>T:p.(Met1?)	LP	start_lost	0.00003368	*GRM6*	Glutamate receptor activity detection of visible light, detection of light stimulus involved in visual perception, retina development in camera-type eye, positive regulation of calcium ion import across plasma membrane
	NM_002332.2:c.1415G>A:p.(Arg472Gln)	VUS	missense_variant&splice_region_variant	0.0001351	*LRP1*	Negative regulation of metallopeptidase activity, RNA binding, heparan sulfate proteoglycan binding, negative regulation of gene expression, regulation of extracellular matrix disassembly, negative regulation of Wnt signaling pathway
**OFT-00223**	NM_017758.3:c.952C>A:p.(Pro318Thr)	VUS	missense_variant	0.00001068	*ALKBH5*	mRNA N6-methyladenosine dioxygenase activity, response to hypoxia, mRNA processing, mRNA export from nucleus, regulation of translation, oxidative single-stranded RNA demethylation, mRNA destabilization, oxidative RNA demethylase activity
	NM_206933.2:c.15172T>C:p.(Phe5058Leu)	VUS	missense_variant	0.00003185	*USH2A*	Photoreceptor inner segment, photoreceptor connecting cilium, neuronal cell body, collagen binding, visual perception, photoreceptor cell maintenance, sensory perception of light stimulus, extracellular region, USH2 complex
**OFT-00253**	NM_025114.3:c.6791A>G:p.(Lys2264Arg)	VUS	missense_variant	0.00006424	*CEP290*	Protein transport, eye photoreceptor cell development, positive regulation of DNA-templated transcription, extracellular region, photoreceptor connecting cilium, camera-type eye development, non-motile cilium assembly
	NM_013291.2:c.3128C>T:p.(Pro1043Leu)	VUS	missense_variant	0.00001200	*CPSF1*	mRNA 3’-UTR AU-rich region binding, mRNA polyadenylation, co-transcriptional RNA 3’-end processing, cleavage and polyadenylation pathway, mRNA cleavage and polyadenylation specificity factor complex, nucleus, mRNA polyadenylation
	NM_005529.6:c.3346G>A:p.(Gly1116Ser)	VUS	missense_variant	0.00005501	*HSPG2*	Collagen-containing extracellular matrix, amyloid-beta binding, calcium ion binding, extracellular matrix structural constituent conferring compression resistance, collagen V binding, angiogenesis, response to hypoxia, positive regulation of endothelial cell proliferation, inflammatory response, negative regulation of angiogenesis, extracellular region, extracellular space, extracellular exosome
	NM_003803.3:c.4580G>T:p.(Gly1527Val)	VUS	missense_variant	0.000008027	*MYOM1*	Structural constituent of muscle, extraocular skeletal muscle development, positive regulation of gene expression, positive regulation of protein secretion, striated muscle myosin thick filament, M band
	NM_003803.3:c.3032T>C:p.(Val1011Ala)	VUS	missense_variant	0	*MYOM1*	Structural constituent of muscle, extraocular skeletal muscle development, positive regulation of gene expression, positive regulation of protein secretion, striated muscle myosin thick filament, M band
	NM_033282.3:c.1411_1412insT:p.(Ser473fs)	VUS	frameshift_variant	0.0009697	*OPN4*	11-cis retinal binding, G-protein-coupled photoreceptor activity, visual perception, phototransduction, optokinetic behavior, detection of visible light, regulation of circadian rhythm, retina development in camera-type eye, photoreceptor disc membrane, phototransduction, cellular response to light stimulus
	NM_003247.3:c.799G>A:p.(Glu267Lys)	VUS	missense_variant	0.0002277	*THBS2*	Extracellular matrix structural constituent, calcium ion binding, protein binding, negative regulation of angiogenesis, positive regulation of synapse assembly, extracellular region, collagen-containing extracellular matrix
**OFT-00268**	NM_033225.5:c.9089C>G:p.(Pro3030Arg)	VUS	missense_variant	0	*CSMD1*	Male gonad development, female gonad development, oviduct epithelium development
	NM_033225.5:c.7050T>A:p.(Ser2350Arg)	VUS	missense_variant	0.00001019	*CSMD1*	Male gonad development, female gonad development, oviduct epithelium development
**OFT-00332**	NM_173728.3:c.380C>G:p.(Pro127Arg)	VUS	missense_variant	0.00001197	*ARHGEF15*	Guanyl-nucleotide exchange factor activity, GTPase activator activity, retina vasculature morphogenesis in camera-type eye, regulation of postsynapse assembly, negative regulation of synapse maturation, dendrite, postsynapse, glutamatergic synapse
	NM_000186.3:c.481G>T:p.(Ala161Ser)	VUS	missense_variant	0.00009160	*CFH*	Heparan sulfate proteoglycan binding, complement activation, extracellular region, extracellular space, extracellular exosome
	NM_013291.2:c.2383G>A:p.(Glu795Lys)	VUS	missense_variant&splice_region_variant	0	*CPSF1*	mRNA 3’-UTR AU-rich region binding, mRNA polyadenylation, co-transcriptional RNA 3’-end processing, cleavage and polyadenylation pathway, mRNA cleavage and polyadenylation specificity factor complex, nucleus, mRNA polyadenylation
	NM_005529.6:c.4078A>G:p.(Asn1360Asp)	VUS	missense_variant	0.0001388	*HSPG2*	Collagen-containing extracellular matrix, amyloid-beta binding, calcium ion binding, extracellular matrix structural constituent conferring compression resistance, collagen V binding, angiogenesis, response to hypoxia, positive regulation of endothelial cell proliferation, inflammatory response, negative regulation of angiogenesis, extracellular region, extracellular space, extracellular exosome
	NM_002332.2:c.11930C>T:p.(Ser3977Leu)	VUS	missense_variant	0.0003749	*LRP1*	Negative regulation of metallopeptidase activity, RNA binding, heparan sulfate proteoglycan binding, negative regulation of gene expression, regulation of extracellular matrix disassembly, negative regulation of Wnt signaling pathway
	NM_201269.2:c.1366A>T:p.(Thr456Ser)	VUS	missense_variant	0	*ZNF644*	RNA polymerase II cis-regulatory region sequence-specific DNA binding, DNA-binding transcription factor activity, RNA polymerase II-specific, transcription corepressor binding, regulation of transcription by RNA polymerase II, nucleus, DNA-binding transcription factor activity
**OFT-00403**	NM_006292.3:c.942C>T:p.(Ile314Ile)	VUS	synonymous_variant	0.0001450	*TSG101*	DNA binding, transcription corepressor activity, ubiquitin protein ligase binding, negative regulation of transcription by RNA polymerase II, regulation of cell growth, extracellular transport, negative regulation of epidermal-growth-factor-activated receptor activity, negative regulation of cell population proliferation
**OFT-00429**	NM_013281.3:c.325T>G:p.(Leu109Val)	VUS	missense_variant	0.0001205	*FLRT3*	Fibroblast growth factor receptor binding, axon guidance, synapse assembly, fibroblast growth factor receptor signaling pathway, neuron projection development, extracellular matrix, extracellular space
	NM_005529.6:c.742_744delCTT:p.(Leu248del)	VUS	conservative_inframe_deletion	0	*HSPG2*	Collagen-containing extracellular matrix, amyloid-beta binding, calcium ion binding, extracellular matrix structural constituent conferring compression resistance, collagen V binding, angiogenesis, response to hypoxia, positive regulation of endothelial cell proliferation, inflammatory response, negative regulation of angiogenesis, extracellular region, extracellular space, extracellular exosome
	NM_005529.6:c.738delT:p.(Leu247fs)	LP	frameshift_variant	0	*HSPG2*	Collagen-containing extracellular matrix, amyloid-beta binding, calcium ion binding, extracellular matrix structural constituent conferring compression resistance, collagen V binding, angiogenesis, response to hypoxia, positive regulation of endothelial cell proliferation, inflammatory response, negative regulation of angiogenesis, extracellular region, extracellular space, extracellular exosome
	NM_002337.3:c.298G>A:p.(Gly100Ser)	VUS	missense_variant	0.0003393	*LRPAP1*	Amyloid-beta binding, signaling receptor binding, endoplasmic reticulum-Golgi intermediate compartment, endosome
	NM_004994.2:c.1270C>A:p.(Arg424Ser)	VUS	missense_variant	0	*MMP9*	Metalloendopeptidase activity, serine-type endopeptidase activity, extracellular matrix disassembly, collagen catabolic process, cellular response to reactive oxygen species, negative regulation of intrinsic apoptotic signaling pathway, negative regulation of cation channel activity, collagen-containing extracellular matrix, extracellular exosome, extracellular matrix organization, collagen catabolic process
**OFT-00474**	NM_014907.2:c.925G>A:p.(Ala309Thr)	VUS	missense_variant	0.0007006	*FRMPD1*	Regulation of G-protein-coupled receptor signaling pathway
	NM_005559.3:c.781A>G:p.(Ile261Val)	VUS	missense_variant	0.00009679	*LAMA1*	Collagen-containing extracellular matrix, extracellular matrix structural constituent, glycosphingolipid binding, morphogenesis of an epithelial sheet, neuron projection development, establishment of epithelial cell apical/basal polarity, retinal blood vessel morphogenesis, positive regulation of integrin-mediated signaling pathway, extracellular region, extracellular space, extracellular matrix, collagen-containing extracellular matrix, protein complex involved in cell–matrix adhesion, laminin-1 complex, laminin-3 complex
	NM_152683.3:c.1380T>C:p.(Cys460Cys)	VUS	splice_region_variant&synonymous_variant	0.0008670	*PRIMPOL*	Chromatin binding, DNA-directed DNA polymerase activity, DNA primase activity, mitochondrial DNA replication, synthesis of RNA primer, replication fork processing
**OFT-00477**	NM_006292.3:c.942C>T:p.(Ile314Ile)	VUS	synonymous_variant	0.0001450	*TSG101*	DNA binding, transcription corepressor activity, ubiquitin protein ligase binding, negative regulation of transcription by RNA polymerase II, regulation of cell growth, extracellular transport, negative regulation of epidermal-growth-factor-activated receptor activity, negative regulation of cell population proliferation
**OFT-00506**	NM_001853.3:c.1511C>T:p.(Pro504Leu)	VUS	missense_variant	0.00006417	*COL9A3*	Extracellular matrix structural constituent conferring tensile strength, protein homodimerization activity, extracellular region, collagen-containing extracellular matrix, collagen type IX trimer, extracellular matrix organization, extracellular space
**OFT-00546**	NM_000350.2:c.6148G>C:p.(Val2050Leu)	VUS	missense_variant&splice_region_variant	0.002857	*ABCA4*	Retinoid binding, 11-cis retinal binding, all-trans retinal binding, retinol transmembrane transporter activity, ABC-type transporter activity, visual perception, phototransduction, visible light, retinol transport, retinal metabolic process, photoreceptor cell maintenance, photoreceptor outer segment, photoreceptor disc membrane, rod photoreceptor disc membrane, ATPase-coupled intramembrane lipid transporter activity
	NM_198576.3:c.2737G>A:p.(Val913Met)	VUS	missense_variant	0.0001489	*AGRN*	Collagen-containing extracellular matrix, extracellular matrix structural constituent, laminin binding, heparan sulfate proteoglycan binding, clustering of voltage-gated sodium channels, positive regulation of transcription by RNA polymerase II, extracellular region, collagen-containing extracellular matrix, extracellular exosome, neuromuscular junction development
	NM_013281.3:c.1135G>A:p.(Gly379Arg)	VUS	missense_variant	0.00001594	*FLRT3*	Fibroblast growth factor receptor binding, axon guidance, synapse assembly, fibroblast growth factor receptor signaling pathway, neuron projection development, extracellular matrix, extracellular space
	NM_000426.3:c.6880G>T:p.(Val2294Leu)	VUS	missense_variant	0	*LAMA2*	Maintenance of blood–brain barrier, structural molecule activity, extracellular matrix structural constituent, axon guidance, positive regulation of integrin-mediated signaling pathway, extracellular region, collagen-containing extracellular matrix, protein complex involved in cell-matrix adhesion
	NM_005560.4:c.11063G>A:p.(Gly3688Glu)	VUS	missense_variant	0.000004051	*LAMA5*	Collagen-containing extracellular matrix, integrin binding, extracellular matrix structural constituent, morphogenesis of a polarized epithelium, integrin-mediated signaling pathway, morphogenesis of embryonic epithelium, regulation of epithelial cell proliferation, postsynapse organization, extracellular region, synaptic cleft, collagen-containing extracellular matrix, extracellular exosome, extracellular matrix of synaptic cleft, laminin-5 complex, laminin-10 complex, laminin-11 complex
	NM_000428.2:c.3998G>C:p.(Gly1333Ala)	VUS	missense_variant	0	*LTBP2*	Supramolecular fiber organization, collagen-containing extracellular matrix, extracellular matrix structural constituent, calcium ion binding, growth factor binding, transforming growth factor beta receptor signaling pathway, protein secretion, extracellular region, extracellular space, extracellular matrix, collagen-containing extracellular matrix, extracellular exosome, supramolecular fiber organization
**OFT-00586**	NM_001080512.2:c.1425G>C:p.(Leu475Phe)	VUS	missense_variant	0.00003186	*BICC1*	RNA binding, determination of left/right symmetry, negative regulation of canonical Wnt signaling pathway
	NM_001206955.1:c.2128G>A:p.(Gly710Arg)	VUS	missense_variant	0.00004244	*CNTN4*	Neuron cell–cell adhesion, nervous system development, axonogenesis, axon guidance, axonal fasciculation, neuron projection development, negative regulation of neuron differentiation, regulation of synaptic plasticity, extracellular region, plasma membrane, axon
	NM_000428.2:c.1487G>A:p.(Gly496Asp)	VUS	missense_variant	0.0003103	*LTBP2*	Supramolecular fiber organization, collagen-containing extracellular matrix, extracellular matrix structural constituent, calcium ion binding, growth factor binding, transforming growth factor beta receptor signaling pathway, protein secretion, extracellular region, extracellular space, extracellular matrix, collagen-containing extracellular matrix, extracellular exosome, supramolecular fiber organization
	NM_006292.3:c.307G>A:p.(Val103Ile)	VUS	missense_variant	0.00001916	*TSG101*	DNA binding, transcription corepressor activity, ubiquitin protein ligase binding, negative regulation of transcription by RNA polymerase II, regulation of cell growth, extracellular transport, negative regulation of epidermal-growth-factor-activated receptor activity, negative regulation of cell population proliferation
**OFT-00601**	NM_033225.5:c.5600C>G:p.(Pro1867Arg)	VUS	missense_variant	0	*CSMD1*	Male gonad development, female gonad development, oviduct epithelium development
**OFT-00710**	NM_000138.4:c.8195G>C:p.(Ser2732Thr)	VUS	missense_variant	0	*FBN1*	Collagen-containing extracellular matrix, integrin binding, extracellular matrix structural constituent, extracellular matrix constituent conferring elasticity, sequestering of BMP in extracellular matrix, sequestering of TGFbeta in extracellular matrix, camera-type eye development, embryonic eye morphogenesis, post-embryonic eye morphogenesis, cellular response to transforming growth factor beta stimulus, anatomical structure morphogenesis
	NM_000428.2:c.2512G>A:p.(Val838Met)	VUS	missense_variant	0.00002578	*LTBP2*	Supramolecular fiber organization, collagen-containing extracellular matrix, extracellular matrix structural constituent, calcium ion binding, growth factor binding, transforming growth factor beta receptor signaling pathway, protein secretion, extracellular region, extracellular space, extracellular matrix, collagen-containing extracellular matrix, extracellular exosome, supramolecular fiber organization
	NM_206933.2:c.9776T>C:p.(Val3259Ala)	VUS	missense_variant	0	*USH2A*	Photoreceptor inner segment, photoreceptor connecting cilium, neuronal cell body, collagen binding, visual perception, photoreceptor cell maintenance, sensory perception of light stimulus, extracellular region, USH2 complex

Variants found in each family with its classification, functional annotation, frequencies in gnomAD global population and main pathways related to each gene affected.
